# Extensive shift in placental transcriptome profile in preeclampsia and placental origin of adverse pregnancy outcomes

**DOI:** 10.1038/srep13336

**Published:** 2015-08-13

**Authors:** Siim Sõber, Mario Reiman, Triin Kikas, Kristiina Rull, Rain Inno, Pille Vaas, Pille Teesalu, Jesus M. Lopez Marti, Pirkko Mattila, Maris Laan

**Affiliations:** 1 Human Molecular Genetics Research Group, Institute of Molecular and Cell Biology, University of Tartu, Tartu 51010, Estonia; 2 Department of Obstetrics and Gynecology, University of Tartu, Puusepa St. 8, Tartu 51014, Estonia; 3 Women’s Clinic of Tartu University Hospital, Puusepa St. 8, Tartu 51014, Estonia; 4 Institute for Molecular Medicine Finland (FIMM), Tukholmankatu 8, Helsinki FI-00014, Finland

## Abstract

One in five pregnant women suffer from gestational complications, prevalently driven by placental malfunction. Using RNASeq, we analyzed differential placental gene expression in cases of normal gestation, late-onset preeclampsia (LO-PE), gestational diabetes (GD) and pregnancies ending with the birth of small-for-gestational-age (SGA) or large-for-gestational-age (LGA) newborns (n = 8/group). In all groups, the highest expression was detected for small noncoding RNAs and genes specifically implicated in placental function and hormonal regulation. The transcriptome of LO-PE placentas was clearly distinct, showing statistically significant (after FDR) expressional disturbances for hundreds of genes. Taqman RT-qPCR validation of 45 genes in an extended sample (n = 24/group) provided concordant results. A limited number of transcription factors including *LRF, SP1* and *AP2* were identified as possible drivers of these changes. Notable differences were detected in differential expression signatures of LO-PE subtypes defined by the presence or absence of intrauterine growth restriction (IUGR). LO-PE with IUGR showed higher correlation with SGA and LO-PE without IUGR with LGA placentas. Whereas changes in placental transcriptome in SGA, LGA and GD cases were less prominent, the overall profiles of expressional disturbances overlapped among pregnancy complications providing support to shared placental responses. The dataset represent a rich catalogue for potential biomarkers and therapeutic targets.

The placenta is the only mammalian organ that functions during an explicitly restricted timeframe with the sole task to support the early life period of the developing fetus. Its unique endocrine, immunomodulatory and “cancerous” properties mediate rapid implantation, trophoblast invasion, proliferation and differentiation, and the processes of vasculogenesis and angiogenesis. The placenta acts at the maternal-fetal interface and serves two organisms – nutrition, metabolism, growth and development of the fetus, and gestational adaptation of the mother. Impaired implantation, aberrant placental development and placental malfunction represent high risks for pregnancy complications such as miscarriage, preeclampsia characterized by *de novo* hypertension and proteinuria, gestational diabetes, fetal growth restriction or preterm birth^1^. Studies on candidate genes and expression microarrays have convincingly established a link between pregnancy complications and aberrant placental gene expression, reflecting impaired placental ‘health’^2^,^3^,^4^,^5^. Several placental secreted proteins, hormones, mRNAs and miRNAs molecules crossing the maternal-fetal barrier represent specific biomarkers for impaired placental function, as their aberrant expression levels are measurable in the maternal circulation^3^,^6^,^7^,^8^,^9^,^10^. As pregnancy complications represent common disorders concerning up to 20% of all gestations, the explicit understanding of gene expression signatures of the malfunctioning placenta is crucial to pinpoint critically involved genes and mechanisms, and to discover novel biomarkers and therapeutic targets for early prediction and clinical management.

So far, the published RNA sequencing (RNA-Seq) studies on the human placenta have been limited to normal term pregnancies and confined to small sample sizes (5–22 samples)^11^,^12^,^13^. More placental gene expression data is available from microarray-based experiments^14^, but these projects have focused mostly on a specific gestational complication, predominantly preeclampsia. Consequently, there is still an open question, whether each pregnancy complication is characterized by distinct aberrant placental gene expression profiles or whether gestational disturbances also share common molecular signatures reflecting overall impaired implantation, placental development and function.

We aimed at a comprehensive and systematic analysis of placental transcriptomes of 40 samples over a broad range of pregnancy outcomes, utilizing the most up-to-date methodology, RNA-Seq. The study profiles placental differential gene expression signatures in prevalent adverse pregnancy outcomes at term, focusing on maternal late-onset preeclampsia (LO-PE), gestational diabetes (GD), and pregnancies ending with the birth of either small-for-gestational-age (SGA) or large-for-gestational-age (LGA) newborns. As the main outcome, we show that preeclamptic placentas are distinguished from normal placentas and other complications by a prominent shift in expression of a large number of genes. Notably, whereas the change in placental gene expression in SGA, LGA and GD cases was less prominent than in PE, the overall profiles of differential expression overlapped among pregnancy complications. The study data represent a rich catalogue for potential biomarkers and therapeutic targets.

## Results

### Placental RNA-Seq dataset

To characterize the placental transcriptome profile in normal compared to complicated pregnancies, we performed RNA-Seq for 40 placental samples taken at delivery and covering multiple well-defined pregnancy outcomes at term. The placental samples represented cases of normal pregnancy (NORM) and gestational complications of the mother (severe late preeclampsia, PE; gestational diabetes, GD) or the newborn (small-for-gestational age, SGA; large-for-gestational age, LGA) (n = 8/group; gestational age > 37 weeks; Table 1). The ratio between male and female placentas, as well as between vaginal delivery/caesarean-section was 19 to 21.

Total RNA extracted from placental samples and depleted of ribosomal RNA was subjected to RNA-Seq (Illumina HiSeq 2000). After filtering, each individual transcriptome yielded, on average 37.7 million 46-base pair paired-end reads (range: 25.2–50.5 million; Supplementary Fig. S1). The reads were aligned to the human genome (version GRCh37) with an average success rate of 83.4% (range: 76.5–87.3%). To evaluate the adequacy of the sequencing length and depth, we used 100-base pairs paired-end sequencing for two placental samples. Although it yielded an increased amount of sequences per sample, the fraction of unaligned sequences was higher, reducing the benefit of longer reads (mapped sequence: 57.8%; Supplementary Fig. S1). The complete RNA-Seq dataset over 40 sequenced placental samples was comprised of 123.7 billion aligned bases and in total providing ~680 fold mean coverage over exonic regions (17-fold per sample; Supplementary Fig. S1).

The placenta is a unique organ, and the preparations of placental tissues may contain not only fetal cells, but also cells of maternal origin. We estimated the proportion of RNA originating from maternal nucleated cells (decidual and nucleated blood cells) by examining the comparative expression of the *Xist* transcript in the XY- and XX-placental samples. *Xist* expression was undetectable for 11 of the 19 placental samples representing male offspring and was at marginal level in the rest of XY- (maximum normalized fragment count: 2881) compared to the XX-placentas (median fragment count: 23948; Supplementary Fig. S2). Median estimate for the fraction of RNA originating from maternal cells in XY-placental samples was 0.93% (mean 3.18%). We expect this estimate to be applicable to female samples as well. Overall, the considered quality assessments indicate that the generated placental RNA-Seq dataset is both technically and biologically of high quality.

### The most highly expressed placental genes at term

**Figure 1:**
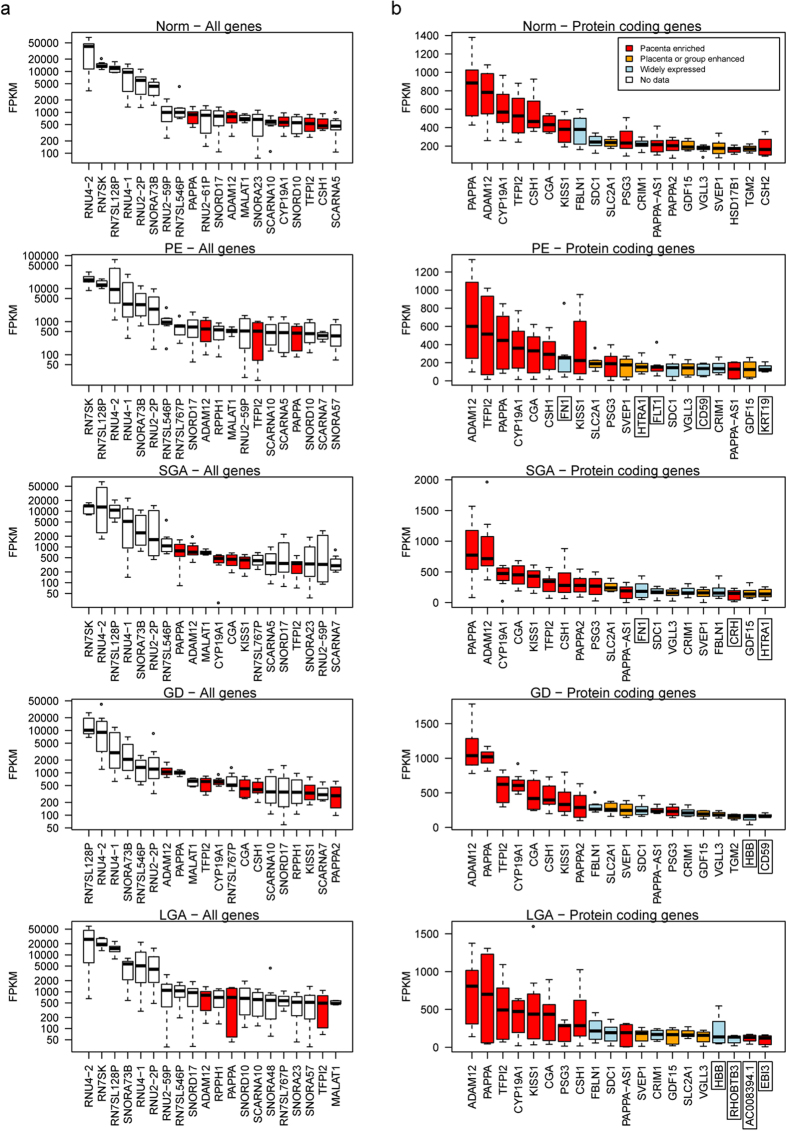
Top 20 transcripts (**(a)** logarithmic scale) and protein coding genes ((**b)** linear scale) with the highest expression in placentas representing normal pregnancy (NORM) and cases of preeclampsia (PE), gestational diabetes (GD), small- and large-for-gestational-age newborns (SGA, LGA) (n = 8/group). Annotation of placental transcripts detected and quantified by the RNA-Seq pipeline was based on ENSEMBL v67 database. Gene expression levels are expressed in FPKM (fragments per kilobase of exons per million mapped fragments) as determined by cufflinks v 2.0.2. Data on the enrichment of gene expression in the placenta compared to other tissues was derived from Protein Atlas v12. Expression profile across tissues for noncoding RNAs was not available. Boxed gene names indicate transcripts ranked among the top-20 highest expressed genes only in specific pregnancy complications, comparative expression values of these transcripts in all studied groups are shown in Supplementary Fig. S3.

We ordered the genes by their median expression level, ranging from 0 to 40,923 FPKM (fragments per kilobase of exons per million mapped fragments) in placentas from normal pregnancies (Fig. 1, Supplementary Data S1). The subsequent analysis included genes with >0 FPKM (n = 22,896) and among these 20,983 genes (15,389 protein coding genes) exhibited expression > 0.1 FPKM (91.6%). Highest expression in both, normal and complicated pregnancies was observed for genes encoding small noncoding RNAs, including small nuclear RNAs contributing to pre-mRNA splicing and processing (e.g. *RN7SK, RNU4-2*, *RNU4-1, RN7SL128P*), small nucleolar RNAs involved in ribosomal RNA processing (e.g. *SNORA73B, SNORD17*) and small RNAs specific to Cajal body, a hallmark of proliferating cells (e.g. *SCARNA10, SCARNA5*) (Fig. 1a, Supplementary Data S1). Among the highest expressed placental transcripts is a highly conserved mammalian long noncoding RNA *MALAT1*, which regulates expression of genes implicated in cellular motility^15^, endothelial cell function and angiogenesis^16^. Its placental expression has not been previously studied.

The most abundantly transcribed placental protein coding genes display a highly organ-specific expression that is robustly captured in our dataset (Fig. 1b, Supplementary Data S1). Several of them encode for hormones supporting the role of the placenta as an endocrine organ important for communication and signaling. The majority of the top-20 placenta-expressed protein coding genes are implicated specifically in placental function and pregnancy outcome: *PAPPA* (encoding pregnancy-associated plasma protein A), *ADAM12* (ADAM metallopeptidase domain 12), *CYP19A1* (estrogen synthase)*, CSH1* and *CSH2* (placental lactogen), *CGA* (hCG alpha subunit), *KISS1* (kisspeptin) and *PSG3* (placenta-specific glycoprotein 3). *TFPI2* (tissue factor pathway inhibitor 2) represents the most highly transcribed imprinted gene (maternally expressed) in the placenta with its expression restricted to the syncytiotrophoblast^17^ and contributing to trophoblast differentiation and the maintenance of intervillous blood flow. As an indication of the overall altered transcriptome, the most highly expressed (top-20) placental genes in pregnancy complications included also a few group-specific genes (n = 10 across all pathologies; Fig. 1b, Supplementary Fig. S3).

### Confounding variables

We addressed maternal, fetal/pregnancy and delivery-related potential confounding variables for the placental transcript profile of all (n = 40) samples. The studied parameters affected the placental gene expression of a limited number of genes. A statistically significant effect (FDR < 0.1) of the delivery mode (vaginal/caesarian section) and labor onset was detected on the expression level of 27 and 28 genes, respectively (Table 2; Supplementary Data S2). As the majority of caesarian-sections are performed to pregnancies without onset of labor, the effect of these parameters cannot be explicitly distinguished. Several genes identified in association with initiated labor are modulators of the progesterone and estrogen function, such as *KLF9*^18^ encoding a progesterone receptor co-regulator; *ID4*, involved in estrogen signaling^19^ and *FKBP5* interacting with functionally mature progesterone receptor complexes^20^. Functional profiling implemented in g:Profiler^21^ revealed significant enrichment of genes down-regulated with the onset of labor to belong to the TGF-beta signaling pathway (KEGG:04350; P = 2.57 × 10^−4^; Supplementary Data S2).

Among the fetal parameters, the most significant confounders for placental gene expression were the offspring’s gender (FDR < 0.1: 20 sex chromosomal genes) and placental weight (FDR <0.1: 16 genes). Functional enrichment analysis resulted in the pathway ‘histone demethylase activity’ (GO:0032452; P = 1.81 × 10^−5^), indicating gender-specific differences in placental epigenetic processes. Interestingly, among the top genes exhibiting significant negative correlation in expression level with placental weight, *LEP* (encoding leptin), *HTRA4* and *LYN* are known as biomarkers for preeclampsia^2^,^5^. Among the genes exhibiting statistical differences in expression levels in association with infant’s birth weight and gestational age at delivery, we detected a novel endothelial scaffold molecule *SASH1*^22^ and estrogen-receptor co-regulator *NRIP1* (*RIP140*), a key modulator of energy homeostasis^23^,^24^. No significantly enriched biological pathways were identified for pregnancy-specific variables. Neither newborn length nor the maternal parameters (age, BMI, parity and weight gain during pregnancy) influenced the placental gene expression in our samples (Table 2; Supplementary Data S2).

### Preeclamptic placentas are outliers and exhibit major transcriptional perturbations

**Figure 2:**
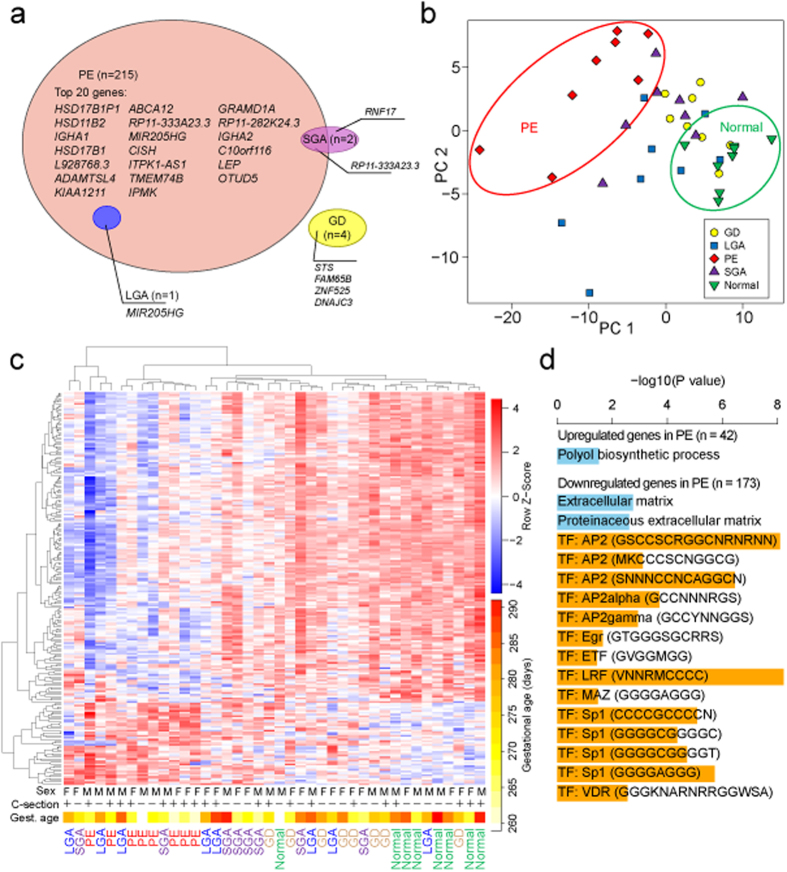
Differential gene expression in cases of preeclampsia (PE), gestational diabetes (GD), cases of small- and large-for-gestational-age newborns (SGA, LGA) compared to normal pregnancy (Normal) based on RNA-Seq profiling of 40 term placental samples (n = 8/group) (**a**) Venn diagram showing differentially expressed genes in each pathology group supported by stringent statistical significance criteria (DESeq: FDR < 0.1 and DESeq2: FDR < 0.05). (**b**) Principal component analysis (PCA, the two first components are plotted) and (**c**) hierarchical clustering based on transformed read counts of 220 differentially expressed genes across pregnancy complications. The gene expression levels were subjected to variance stabilizing transformation in DESeq and standardized by subtracting the mean expression across all samples from its value for a given sample and then dividing by the standard deviation across all the samples. This scaled expression value, denoted as the row Z-score, is plotted in red-blue color scale with red indicating increased expression and blue indicating decreased expression. Hierarchical clustering of genes (rows) and samples (columns) was based on Pearson’s correlation. Hierarchical clustering trees are shown for the analyzed samples (top) and genes (left). For each sample are shown newborn sex (M, male; F, female), delivery by caesarean section (+/–) and gestational age at birth plotted in white-yellow-red color scale (white < 260, red > 290 gestational days). (**d**) Significantly enriched categories among the significantly differentially expressed genes in PE (n = 215) from gene set enrichment analysis in g:Profiler. Horizontal bars indicate significance. Blue bars represent GO terms, orange bars represent transcription factor binding sites.

We addressed differential expression of the term placental transcriptome in maternal late-onset preeclampsia (LO-PE) and gestational diabetes (GD), and affected fetal growth (small- and large- for-gestational age newborns; SGA, LGA) in comparison to normal (NORM) pregnancies (n = 8/group). Among the studied pregnancy complications, PE placentas were distinguished by a major shift in the expression profile of hundreds of genes (Fig. 2a; Supplementary Data S3; Supplementary Table S1). Differential expression of 215 genes matched the significance criteria applied in this study (DESeq: false discovery rate (FDR) < 0.1, DESeq2: FDR < 0.05; Supplementary Table S1; details in Materials and Methods). Notably, 80% (n = 173) of the differentially expressed genes in LO-PE placentas showed significantly lower transcript levels compared to the NORM group (Fig. 2, Supplementary Data S3). The top list contained known loci implicated in placental function and PE, such as the up-regulated *LEP* and down-regulated

*HSD11B2* and *HSD17B1* genes^2^,^5^. Only a few transcripts exhibited statistically significant placental differential expression in other complications: GD (*STS, FAM65B, ZNF525, DNAJC3*), SGA (*RNF17, RP11-333A23.3*) and LGA (*MIR205HG*). Principal component (PCA) analysis and hierarchical clustering clearly separated LO-PE from NORM placental samples, whereas the cluster of GD placentas overlapped with the NORM group (Fig. 2b,c). The placental gene expression profile in the SGA and LGA cases represented a more scattered landscape partially overlapping with the PE and GD groups. To assess the contribution of potential confounding variables on the hierarchical clustering of the data, the generated heatmap was evaluated in the context of the newborn’s sex, gestational age and mode of delivery of each placental sample (Fig. 2c). No noticeable effect on gene expression patterns was observed for newborn sex and delivery mode. As the delivery date in PE pregnancies in this study was on average 12.5 days earlier than that in the normal gestation group (Table 1), we cannot fully rule out a potential confounding effect of gestational age. However, as no gestational age dependent clustering either within the PE group or outside of it was observed, we concluded that the identified differential expression profile in our dataset reflects primarily the disturbed placental transcriptome in preeclamptic pregnancies.

### Regulation of differentially expressed genes in preeclampsia is orchestrated by a set of transcription factors

To further functionally characterize the altered transcriptome profile in preeclamptic placentas, we performed gene set enrichment analysis for the identified significantly under- and overexpressed genes compared to normal pregnancy (RNA-Seq: n = 173 and n = 42, respectively; Supplementary Data S3). For the down-regulated transcripts, the most pronounced enrichment was detected for the binding sites of a group of transcription factors (TF) predicted to regulate the analyzed genes (Fig. 2d; Supplementary Table S2). We observed strong enrichment for the presence of binding sites for *AP2* (P = 8.14 × 10^−9^; 115/173 genes), *SP1* (P = 8.23 × 10^−6^; 95/173 genes) and *LRF* (P = 5.96 × 10^−9^; 140/173 genes) (Supplementary Data S4). Promoter regions of 77 genes (44.5%) with highly significant decrease in placental expression in PE contain potential response elements for all three TFs. In contrast, functional profiling of biological pathways affected in PE placentas only detected a moderate enrichment of the genes involved in extracellular matrix (down-regulated transcripts; P = 1.76 × 10^−3^) and polyol biosynthesis (up-regulated genes; P = 0.03). These data make it tempting to propose the hypothesis that the underlying major disturbance in the transcriptional profile in preeclampsia may be attributable to changes in transcriptional regulation by a limited number of TFs.

### Confirmation of RNA-Seq results by quantitative PCR

**Figure 3:**
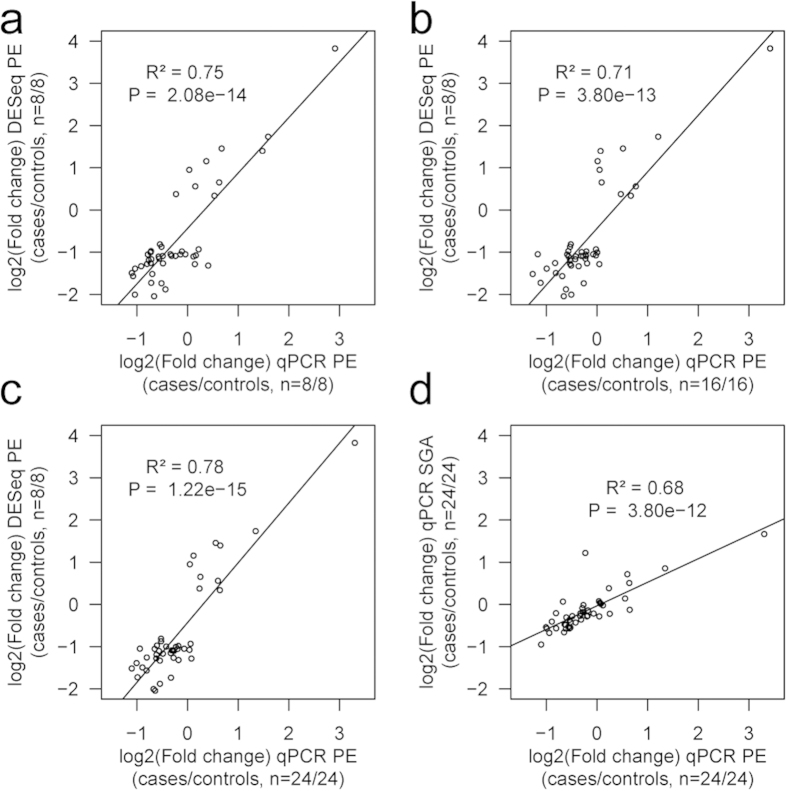
Estimated gene expression log2(fold change) of the 45 tested placental genes in preeclamptic placentas (PE) compared to normal gestation (NORM) is highly correlated between the RNA-Seq and Taqman RT-qPCR datasets. The correlation with RNA-Seq results (Y-axis) holds for the (**a**) technical replicate by the RT-qPCR performed in the discovery samples (PE, n = 8; NORM, n = 8), (**b**) for the biological replicate by RT-qPCR using an independent placental sample-set (PE, n = 16; NORM, n = 16) and (**c**) for the combined RT-qPCR data of discovery and follow-up samples (PE, n = 24; NORM, n = 24) (X-axis). (**d**) The estimated gene expression log2(fold change) of the 45 placental genes subjected to Taqman RT-qPCR in small-for-gestational-age (SGA, n = 24; Y-axis) cases compared to normal gestation (NORM, n = 24) is correlated with gene expression shifts in PE placentas (n = 24; X-axis). Note the difference in slope of the regression line due to more prominent fold changes of all genes in PE compared to SGA. Each dot represents one tested gene and the plots present linear regression lines, *P* values and correlation coefficients (R^2^).

The Taqman RT-qPCR (reverse transcription-qPCR) technical replicate assays performed for the discovery samples (PE, n = 8 *vs* NORM, n = 8) were highly consistent with RNA-Seq data. The correlation between the estimated log2(fold change) of the 45 tested genes in preeclamptic compared to normal placentas was R^2 ^= 0.75 (linear regression, *P *= 2.08 × 10^−14^; Fig. 3a). RT-qPCR in an expanded sample-set (PE, n = 24 *vs* NORM, n =24; Table 1) further confirmed the altered gene expression in preeclampsia with concordant effect direction for 42 of 45 assessed genes (Supplementary Table S3). The estimated log2(fold change) in transcript levels significantly correlated with the RNA-Seq dataset (R^2 ^= 0.78; *P *= 1.22 × 10^−15^; Fig. 3b,c). Four of the top confirmed loci (Supplementary Table S3) have been previously implicated in PE (*FLT1, HSD17B1, DLX4, ADM*). Other confirmed genes point to altered regulation of epigenetic (*DOT1L, TET3*), transcriptional (*ZNF469*) and apoptotic (*RELL2*) mechanisms as well as disturbances in the immune (*IGHA1*) and endocrine-metabolic systems (*HSD17B1, ADM, GDPD5, MC1R*). Multiplicity of affected biological systems supports the systematic malfunction of the placental genome in preeclampsia.

**Figure 4:**
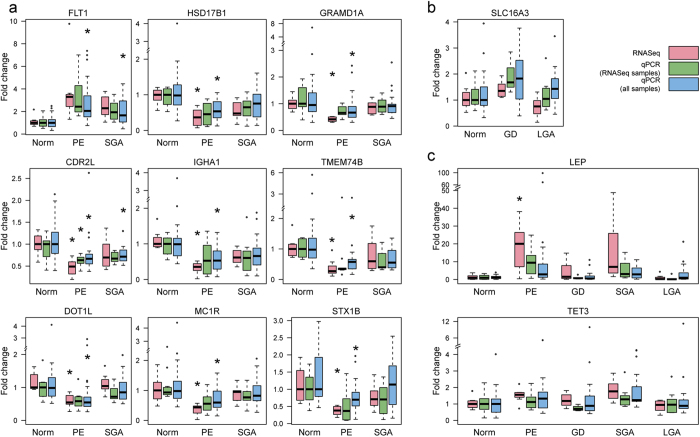
Examples of technical replicates from the experimental validation of differentially expressed placental genes in pregnancy complications. Plots represent gene expression fold changes relative to the median value of the normal pregnancy samples (treated as the reference level = 1) in the discovery RNA-Seq dataset (pink; n = 8 samples/group), in the validation by Taqman RT-qPCR (green; n = 8 samples/group) and in the complete Taqman RT-qPCR dataset (blue; n = 24 samples/group). (**a**) Genes with the highest statistical significance in gene expression shift in pre-eclamptic (PE) placentas (RT-qPCR: FDR <0.05; Supplementary Table S3) show concordant effect directions in the PE and small-for-gestational-age (SGA) groups. (**b**) Placental expression of *SLC16A3* in gestational diabetes (GD) and large for gestational age (LGA) groups. (**c**) *LEP* and *TET3* show elevated transcript level in PE and SGA placentas compared to other pregnancy outcomes. Asterisks (*) indicate differential expression meeting the statistical significance criteria either for the RNA-Seq (DESeq: FDR < 0.1, DESeq2: FDR < 0.05) or RT-qPCR (FDR < 0.05) datasets. *CDR2L*, cerebellar degeneration-related protein 2-like*; DOT1L*, DOT1-like histone H3K79 methyltransferase*; FLT1*, fms-related tyrosine kinase 1; *GRAMD1A*, GRAM domain containing 1A; *HSD17B1*, hydroxysteroid (17-beta) dehydrogenase 1; *IGHA1*, immunoglobulin heavy constant alpha 1; *LEP, leptin; MC1R*, melanocortin 1 receptor (alpha melanocyte stimulating hormone receptor)*; TET3*, tet methylcytosine dioxygenase 3*; TMEM74B*, transmembrane protein 74B; *SLC16A3*, solute carrier family 16 (monocarboxylate transporter); *STX1B*, syntaxin 1B.

As PE and SGA placentas have been suggested to share common pathophysiology^1^,^5^, we performed RT-qPCR for the 45 PE-related genes also in the SGA samples (extended sample, SGA, n = 24 *vs* NORM, n = 24; Table 1). For 78% of genes (n = 35), the direction of expression alteration was concordant between the PE and SGA placentas (Supplementary Table S3). Although for the majority of genes the PE placentas exhibited more prominent change in transcript levels, the effects in the PE and SGA groups were highly correlated (R^2^ =0.68, linear regression *P *= 3.80 × 10^−12^; Fig. 3d). The top-loci *TMEM74B, FLT1, CDR2L* showed significant differential expression in both, PE and SGA placentas (FDR < 0.05; Fig. 4a).

Three genes (*FAM65B, STS, SLC16A3*) were followed-up by RT-qPCR only in the GD and LGA placental samples (n = 24/group; Table 1). The GD group had significant, 1.5-fold increased placental expression of *SLC16A3*, encoding a lactate transporter MCT4 (monocarboxylate transporter 4) (FDR = 0.065; Fig. 4b, Supplementary Table S3). Consistent with the pathophysiology of GD, it is known that metabolic monocarboxylates can compensate glucose as an energy source in diabetes and fasting^25^.

Two genes were investigated for differential expression in all groups. The *LEP* gene encodes leptin, an acknowledged marker for several pregnancy complications and was among the top PE-related genes in our placental RNA-Seq data (Supplementary Data S3). RT-qPCR confirmed a trend for higher placental transcript levels of *LEP* in PE (fold change = 10.02, *P *= 0.057) and SGA (fold change = 3.08, P = 0.033), whereas no difference was detected in the LGA and GD groups (Fig. 4c, Supplementary Table S3). *TET3* encodes TET methylcytosine dioxygenase 3 that converts 5-methylcytosine to 5-hydroxymethylcytosine and has a recently discovered role in the epigenetic regulation of embryonic development. In the RNA-Seq data, *TET3* showed a trend for differential expression in the SGA placentas (DESeq: *P *= 0.0029; DESeq2: *P *= 0.00035). RT-qPCR confirmed 1.7- and 1.5-fold increased placental expression of *TET3* in SGA and PE, respectively (FDR < 0.1; Fig. 4c, Supplementary Table S3).

### Pregnancy complications share signatures of placental malfunction

**Figure 5:**
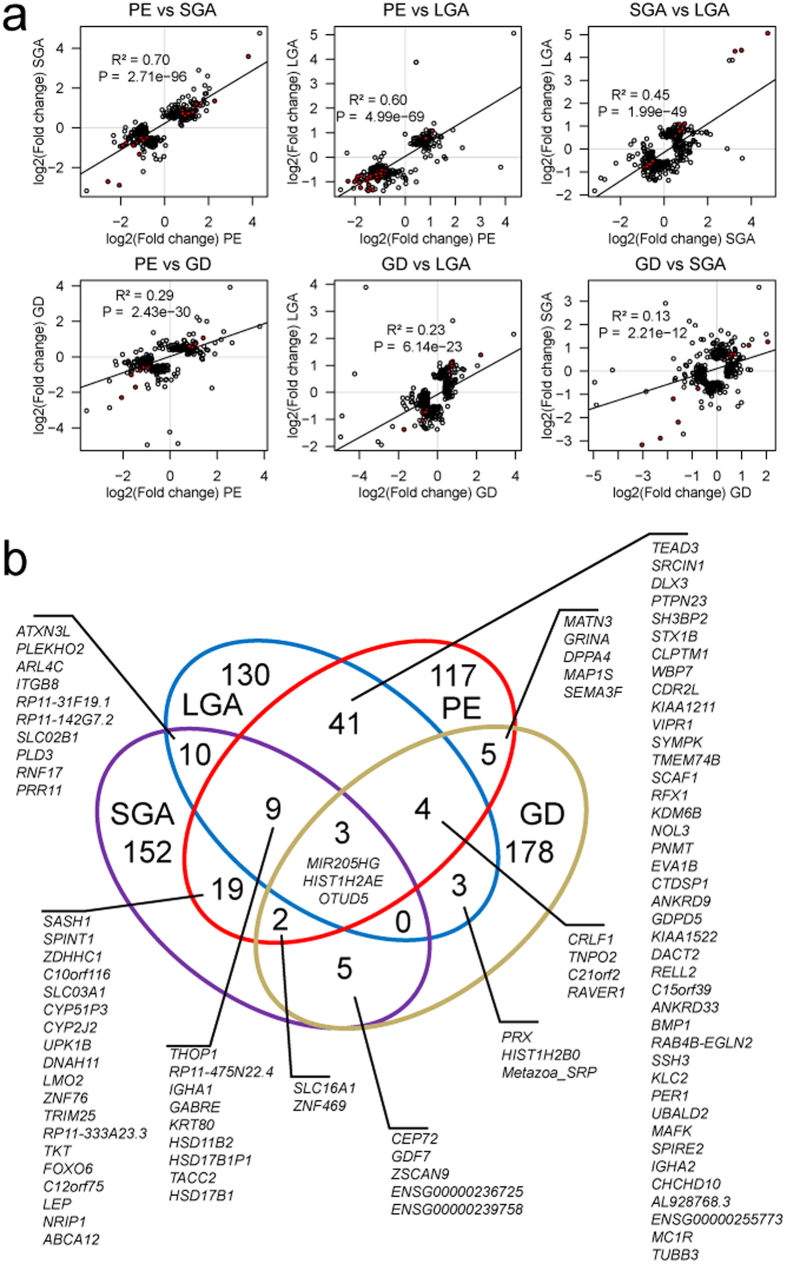
High concordance in placental gene expression changes in alternative scenarios of complicated compared to normal pregnancy. (**a**) Correlation plots for the fold changes of the highest ranked genes in the differential expression testing in each pregnancy complication compared to normal pregnancy (DESeq analysis). For each pairwise analysis of gestational complications, the lists of top-200 genes (circles) were united and plotted at the x,y-plane, where the axes correspond to the log2(fold changes) in the two groups. Red circles represent genes, which are shared between the top gene lists. The linear regression line along with correlation coefficient R^2^ and statistical significance is given. (**b**) Venn diagram for the shared fraction of the highest ranked genes in differential expression testing in alternative pregnancy complications. The number and gene list in each intersection are given. PE, preeclampsia, GD, gestational diabetes, SGA and LGA, small- and large-for-gestational-age newborns.

In order to investigate potential shared placental pathophysiology of pregnancy complications, we compared the estimated differential expression effects for the top-200 genes from the RNA-Seq analysis of LO-PE, GD, SGA and LGA placentas, irrespective of statistical significance. The highest concordance in gene expression disturbances compared to normal pregnancy was detected for SGA and LO-PE placentas, correlation between the log2(fold change) was R^2 ^= 0.70 (linear regression, *P *= 2.71×10^−96^; Fig. 5a). All genes with increased placental transcript levels in PE were concordantly up-regulated in SGA and only a few genes down-regulated in PE were not affected in SGA. Strong correlation between the effects of top-200 genes was also identified for LO-PE and LGA (R^2 ^= 0.60; *P *= 4.99 × 10^−69^) placentas. Unexpectedly, we also detected correlation between the expression changes of the top-200 genes in the SGA and LGA placentas compared to normal pregnancy (R^2 ^= 0.45; *P *= 1.99 × 10^−49^), possibly indicative to placental adaptations in unfavorable conditions. Among these, 65 genes exhibited opposite effect directions, including well-acknowledged markers of aberrant placental development (*LEP, FLT, ENG, HTRA4*, *SH3PXD2A*) (Supplementary Table S4). Gene expression profile of top-200 genes in GD placentas exhibited a less pronounced overlap with other pathologies (R^2 ^= 0.13–0.29; *P *≤ 2.21 × 10^−12^; Fig. 5a).

Consistent with the correlation analysis, the highest number of overlapping genes with altered placental expression was detected for the PE and LGA (n = 57/200 top-genes) and the PE and SGA comparisons (n = 33) (hypergeometric test for enrichment, *P *< 4.44 × 10^−16^; Fig. 5b). For SGA and LGA placentas, the top-200 lists contained 22 overlapping genes (12 shared with other pathologies). The top gene-list with altered expression in GD overlapped with other conditions to a lesser extent, but still statistically significantly (PE, n = 14; LGA, SGA, n = 10 genes; P < 4.71 × 10^−4^; Fig. 5b).

### Evidence for transcriptionally distinct subtypes of late-onset preeclampsia

**Figure 6:**
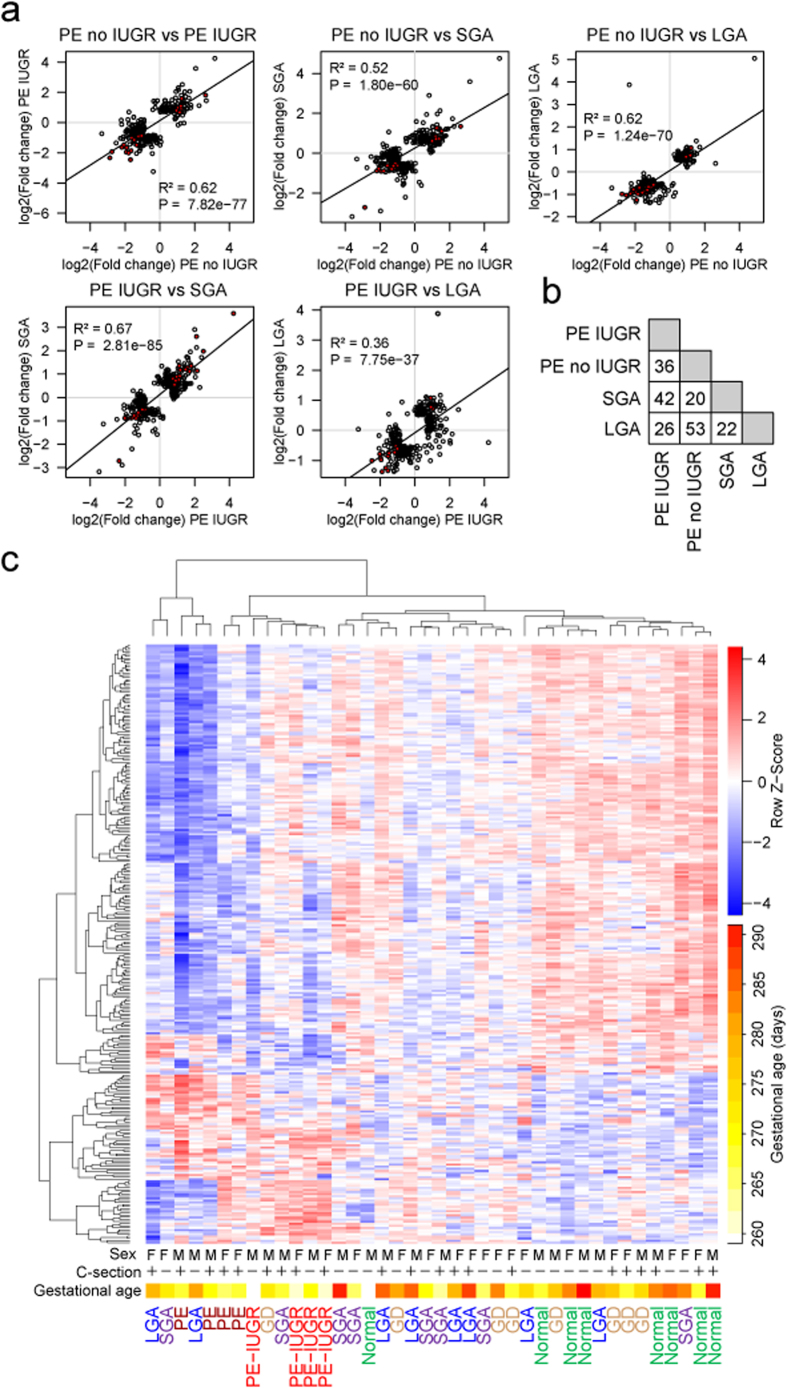
Placentas from the cases of late-onset preeclampsia (PE) with and without concomitant intra-uterine growth restriction (IUGR) exhibit distinct gene expression patterns. (**a**) Correlation plots for the fold changes of the highest ranked genes in the differential expression testing in each pregnancy complication compared to normal pregnancy (DESeq analysis). For each pairwise analysis of gestational complications, the lists of top-200 genes (circles) were united and plotted at the x,y-plane, where the axes correspond to log2(fold changes) in the two groups. Red circles represent genes, which are shared between the top gene lists. The linear regression line along with correlation coefficient R^2^ and statistical significance is given. (**b**) Numbers of shared genes among the top 200 highest ranked transcripts in differential expression testing. Detailed information on the pairwise overlaps among the study groups for the shared top-genes with altered placental expression is provided in Supplementary Fig. S4. (**c**) Hierarchical clustering based on transformed read counts of 283 differentially expressed genes in PE without IUGR, PE with IUGR, SGA, LGA and GD. Gene expression levels were subjected to variance stabilizing transformation in DESeq and standardized by subtracting the mean expression across all samples from its value for a given sample and then dividing by the standard deviation across all the samples. This scaled expression value, denoted as the row Z-score, is plotted in red-blue color scale with red indicating increased expression and blue indicating decreased expression. Hierarchical clustering of genes (rows) and samples (columns) was based on Pearson’s correlation. Hierarchical clustering trees are shown for the analyzed samples (top) and genes (left). For each sample are shown newborn sex (M, male; F, female), delivery by caesarean section (+/–) and gestational age at birth plotted in white-yellow-red color scale (white < 260, red  > 290 gestational days).

Recently, Redman and colleagues have suggested that there are two main placental causes for preeclampsia. PE caused by poor placentation in early pregnancy is frequently accompanied with fetal growth restriction, whereas at term PE may also develop when placental growth reaches its functional limits and is often linked to macrosomy^26^. To further dissect the relationship between LO-PE and fetal growth we divided the PE study sample according to the presence of concomitant intrauterine growth restriction (IUGR). The two subgroups (n =4/group) were separately tested for the differential placental gene expression compared to the normal gestation group (n = 8). We identified 199 and 98 differentially expressed genes in PE without IUGR and PE with IUGR, respectively (Supplementary Data S3). Only 20 genes overlapped between the two LO-PE subtypes with statistically significant alternations in transcript levels. Still, examination of the top 200 highest ranked genes in both analyses revealed substantial correlation in their expressional changes compared to normal pregnancy (R^2 ^= 0.62; *P *= 7.82 × 10^−77^; 36 shared genes among the top-200; Fig. 6a–b). Notably, placental transcriptome profile in cases of LO-PE with IUGR showed the highest correlation with SGA group (R^2 ^= 0.67; *P *= 2.81 × 10^−85^; 42 shared genes; Fig. 6a–b), whereas the LO-PE without IUGR bears the closest similarity to LGA cases (R^2 ^= 0.62; *P *= 1.24 × 10^−70^; 53 shared genes; Fig. 6a–b). Hierarchical clustering analysis based on all 283 genes matching the statistical significance criteria across study groups also separated the transcriptome profiles of LO-PE with and without IUGR, supporting their distinct molecular signatures (Fig. 6c).

## Discussion

Our study aimed at describing the human placental transcriptome and investigating its link to pregnancy complications. We utilized RNA-Seq to profile the placental transcriptome at term for 40 samples representing normal gestation (NORM), late-onset preeclampsia (LO-PE), gestational diabetes (GD), as well as variations in fetal growth defined as small- and large-for-gestational-age newborns (SGA, LGA).

The following aspects were considered for the study design. We elected to perform RNA-Seq on full thickness placental samples in order to adequately capture the biological state of the entire organ. Although our approach sacrificed gene expression information at the level of cell types, it avoided excessively disturbing the cells prior to RNA isolation. Among the PE and SGA pregnancies, the study profiled only severe LO-PE and term SGA cases and thus, drawing parallels to previous studies on mostly early-onset PE (EO-PE) and preterm SGA/intrauterine growth restriction (IUGR) studies has its limitations. To increase the robustness of the analysis, we utilized two software packages DESeq and DESeq2 defining statistically significant differential expression as the intersection of the output of both programs and performed extensive RT-qPCR validation series, which were in good agreement with the RNA-Seq data.

Our study highlights the importance of estrogen and progesterone metabolism in preparation for delivery and timing of parturition. One of the top genes upregulated in the placentas with initiated labor (mostly vaginal delivery) was *KLF9*, encoding a progesterone receptor co-regulator Krüppel-like Factor 9 (Table 2, Supplementary Data S2). This is consistent to the reported delayed parturition in KLF9 knock-out mice^27^ and a recent report showing the contribution of myometrial KLF9 to the triggering of human parturition^28^. We note that a scaffold molecule *SASH1* involved in endothelial cell migration and TLR4 signaling^22^ and *NRIP1*, a transcriptional modulator of the estrogen receptor^23^,^24^ are down-regulated in placentas with advanced gestational age, and propose that their decreased expression along with elevated transcription of *KLF9* may serve as a hallmark of placental maturation and preparation for delivery. The identified placental molecular signatures related to labor initiation may provide therapeutic targets for management of prolonged pregnancy (no labor at 42 gestational weeks). Additionally, inhibition of this pathway may hinder the progression of threatening preterm birth, one of the largest risk factors for perinatal survival and health.

When comparing the XX- and XY-placentas, we detected statistically different expression only for sex chromosomal genes (Table 2, Supplementary Data S2). A meta-analysis of 11 studies based on gene expression microarrays (303 samples) identified 88 autosomal genes differentially expressed between male and female placentas^14^. As the effect sizes for the majority of these genes were small (<1 SD) and close to significance cutoff, it may explain the limitations in replicating these findings in our smaller sample set. No genes were differentially expressed depending on the investigated maternal variables (age, parity, pre-pregnancy BMI, gestational weight gain).

As a major finding, we show that the transcriptome profile of LO-PE placentas is clearly distinguishable from the cases representing normal pregnancies and other gestational complications (Fig. 2; Supplementary Data S3). In total, we report 215 differentially expressed genes according to the stringent statistical significance criteria in our study, and additional several thousands of genes showing a trend of altered gene expression. Differentially expressed genes in LO-PE placentas represent diverse functions and are not confined to specific molecular pathways or biological functional categories. However, the genes down-regulated in PE display evidence of co-regulation by a set of placental transcription factors including *AP2* (activating protein 2), *SP1* (specificity protein 1) and *LRF* (Leukemia/lymphoma-related factor; also known as *ZBTB7A*). The genes encoding AP2alpha and AP2gamma (*TFAP2A*, *TFAP2C*; Fig. 2d) are categorized as ‘tissue enhanced’ as predominantly expressed in the placenta and skin^29^. The accumulated data indicates essential, but distinct roles of *TFAP2A* and *TFAP2C* in human trophoblast differentiation^30^. While *TFAP2C* expression in cytotrophoblast cells is suggested to support lineage identity, proliferation, migration and invasion, *TFAP2A* is expressed predominantly in syncytiotrophoblast regulating the expression of placental hormones. Also *SP1* is known to regulate several essential highly expressed placental genes such as *CGB*^31^, *HSD11B2*^32^ and *CYP19A1*^33^. In contrast, the knowledge on the role of *LRF* in pregnancy is scarce. Only one study has been published^34^, reporting the expression of *LRF* specifically at implantation sites in mouse uterus, low levels of *LRF* in uteri of mice with delayed implantation and a cross-talk of *LRF* with steroid hormones such as progesterone and estradiol.

The majority of previous studies have investigated placental gene expression in EO-PE (<34 gestational weeks) due to its more pronounced course and a significant risk to the prematurely delivered infant or fetal death. We specifically focused on LO-PE (defined ≥ 34 weeks; criterion in this study > 37 weeks), which has received less attention despite its >7-fold higher prevalence compared to EO-PE^35^. There is growing understanding that LO-PE differs from the EO-PE in its etiology. EO-PE has been ascribed to poor placental development and placentation in early pregnancy often in combination with maternal predisposition, whereas LO-PE has been attributed either to reaching placental limits of growth and functional capacity or failure of normal regulation of maternal blood pressure and kidney function^26^,^36^. Also a recent report comparing placental gene expression microarray dataset on EO- and LO-PE supports the scenario that these are at least partly two separate entities^37^. Two studies aiming to detect a ‘Meta signature’ for PE from published placental microarray datasets (mainly EO-PE) identified a few significant genes in common^2^,^38^. Among these, the list of top-300 differentially expressed genes in our RNA-Seq dataset for the LO-PE placentas included upregulated *LEP, FLT, HEXB, HTRA4, SASH1* and *TREM1*, and down-regulated *HSD17B1*. Indicative to the heterogeneity in the conducted studies, several additional ‘Meta signature’ genes^2^,^38^ were detected in our top-gene lists for the placental differential expression in LO-PE with IUGR (increased *INHBA, PAPPA2, BCL6* and decreased *F13A1, FAM101B*), in LO-PE without IUGR (decreased *PGF*), in SGA (increased *QPCT, FSTL3*, *BHLHE40, CRH, IGFBP1* and decreased *GOT1*) and in LGA (decreased *PLEC, PVRL4, RDH13)*. This underlines the importance to conduct placental gene expression studies including cases of several (thoroughly clinically phenotyped) pregnancy outcomes in order to identify shared and distinct molecular signatures. The accumulated evidence for PE supports the scenario of a spectral disorder driven by the deregulation of different molecular pathways^5^,^26^,^36^,^39^,^40^.

Intriguingly, this study detected the highest number of overlapping genes in the lists of top-200 transcripts with altered placental expression in the PE and LGA groups. Although fetal macrosomy has been regarded as one of the factors driving the placenta to its functional maximum^41^, our PE cases did not include any LGA-newborns. However, when the PE placentas were subgrouped according to the fetal growth parameters, it revealed a marked distinction in the altered expression profiles between the LO-PE with and without IUGR. Placental transcriptome of the LO-PE with IUGR cases aligned more closely with the SGA group, whereas cases of LO-PE without IUGR exhibited the highest correlation with the placental gene expression in LGA. We speculate that placental transcriptome may respond similarly to both conditions, either the exhaustion of maternal capacity to support the growing fetus and finally provoking LO-PE, or the fetal macrosomy gradually driving the placental functional capacity to its limits. The established biomarkers for EO-PE such as sFLT and PlGF^7^,^42^ have been shown to perform poorly for predicting LO-PE^43^. The importance of novel biomarkers for LO-PE is underscored by the tendency of LO-PE to affect seemingly healthy mothers with no apparent risk factors such as high BMI or elevated blood pressure. The revealed intricate relationships between LO-PE and fetal growth disturbances highlight the difficulties in identifying prognostic markers truly informative on the risk to develop PE.

Overall, the scale of expressional differences in GD, SGA and LGA placentas compared to the NORM group was less prominent compared to the PE group and only a few genes reached statistical significance. Among the investigated pregnancy complications, GD differed the least from the NORM group, pointing to other causes than altered placental gene expression in this condition. Still, our RNA-Seq dataset provides evidence for the presence of shared molecular signatures of impaired placental function. Despite the small effect sizes, there is a high correlation among the fold changes of the top genes with altered expression levels in PE, SGA and LGA placentas.

## Conclusions

A systematic investigation of the term placental transcriptome across a wide spectrum of pregnancy complications allowed us to outline the common and distinct features of placental gene expression profiles in each condition. The results support placental origin of preeclampsia and provide evidence for the shared molecular signatures of placental malfunction. The generated dataset represents a rich catalogue of potential biomarkers and therapeutic targets to be taken forward to subsequent translational studies aiming at improved management of gestational disturbances.

## Methods

### Ethics statements

The study was approved by the Ethics Review Committee of Human Research of the University of Tartu, Estonia (permissions no 117/9, 16.06.2003; 146/18, 27.02.2006; 150/33, 18.06.2006; 158/80, 26.03.2007; 180/M-15, 23.03.2009) and it was carried out in compliance with the Helsinki Declaration. A written informed consent to participate in the study was obtained from each individual prior to recruitment. All study participants were recruited and the study material was collected at the Women’s Clinic of Tartu University Hospital, Estonia in 2006–2011. All participants were of white European ancestry and living in Estonia. All methods were carried out in accordance with approved guidelines.

### Study groups

The study participants were recruited at the Women’s Clinic of Tartu University Hospital, Estonia in the framework of the REPROgrammed fetal and/or maternal METAbolism (REPROMETA) study. Cases with documented fetal anomalies, chromosomal abnormalities, families with history of inherited diseases and patients with known pre-existing diabetes mellitus, chronic hypertension and chronic renal disease were excluded. The quality of the REPROMETA sampleset has been highlighted in a devoted editorial^44^.

The control group comprised of uncomplicated pregnancies, which resulted in the delivery of a newborn with normal birth weight for its gestational age (NORM; birth-weight between 10^th^–90^th^ centile). Study groups of fetal growth variations comprised of newborns born as small-for-gestational age (SGA, <10^th^ centile) and large-for-gestational age (LGA, >90^th^ centile). The weight centiles for defining SGA and LGA were calculated on the basis of data from Estonian Medical Birth Registry^45^. Newborns were retrospectively assigned as being growth restricted (IUGR; intrauterine growth restriction) if the most recent ultrasound examination demonstrated abnormal Doppler waveforms in the umbilical or middle cerebral artery and/or if newborn’s weight and/or abdominal circumference was <10^th^ percentile while head circumference was >10^th^ percentile. The individuals recruited into SGA and LGA group had no major gestational complications of the mother.

Study groups of maternal pregnancy complications included severe preeclampsia (PE) and gestational diabetes (GD). Severe PE was defined according to the ACOG 2013^46^ criteria. All patients exhibited hypertension (systolic blood pressure ≥160 mmHg and/or diastolic blood pressure ≥110 mmHg). In addition each patient presented with at least one of the symptoms: thrombocytopenia; impaired liver function; new development of renal insufficiency; proteinuria >5 g in 24 hours; new-onset cerebral or visual disturbances. All included PE cases had the onset of the first symptoms: new development of hypertension or proteinuria, after 34 gestational weeks (late-onset PE). The progression of PE to severe form requiring the delivery occurred no earlier than 35 completed weeks. GD was diagnosed when 75 g oral glucose tolerance test performed at 24–28 weeks of gestation revealed either a fasting venous plasma glucose level of >5.1 mmol/l, and/or at 1 h and 2 h plasma glucose level of >10 mmol/l and >8.5 mmol/l glucose, respectively.

The discovery samples for the RNA-Seq experiment comprised of 40 REPROMETA placentas, eight samples per each clinical subgroup (NORM, LO-PE, GD, SGA, LGA) (Table 1). The LO-PE group was comprised of four cases accompanied with IUGR and four cases without IUGR. Gestational age and amount of male and female fetuses was kept as close as possible between and within all study groups. All groups included cases of both vaginal and caesarean section delivery (4/4, 3/5 or 5/3). Taqman RT-qPCR validation experiments were performed using 120 placentas (n = 24/group), comprised of 40 samples analyzed by RNA-Seq (technical replicate) and 80 independent samples (n = 16/group) selected to maximally match the parameters of the discovery set (Table 1).

### Tissue collection, storage and RNA extraction

Placentas (stored at +4 °C) were sampled within 1 h after vaginal delivery or caesarean section. Full-thickness blocks of 2–3 cm were taken from the middle region of the placenta. Collected tissue samples were washed with 1 × PBS to remove contamination of maternal blood, placed immediately into RNAlater solution (Ambion Inc, Life Technologies) and kept at −80 °C until RNA isolation. All samples were collected by the same medical personnel and using identical protocol. Total RNA was extracted from 200–300 mg of homogenized placental tissue using TRIzol reagent (Invitrogen, Life Technologies) and purified with RNeasy MinElute columns (Qiagen, Netherlands) for RNA-Seq or NucleoSpin^®^ II Isolation Kit (Macherey-Nagel GmbH & Co. KG, Düren, Germany) for Taqman RT-qPCR according to the manufacturers’ protocols. Purity level and concentration of isolated total RNA was measured using NanoDrop^®^ ND-1000 UV-Vis spectrophotometer (Thermo Fisher Scientific Inc., USA) and RIN (RNA integrity number) was estimated using Agilent 2100 Bioanalyzer (Agilent Technologies, USA).

### RNA-Seq

High quality DNA-free total RNA (5 μg) was used for rRNA depletion (Ribo-Zero™ rRNA Removal Kit, Epicentre) and library preparation with Nextera™ Technology (Illumina) total RNA sequencing was performed on Illumina HiSeq2000 with 46 bp paired end reads (101 bp for two samples). Initial data analysis and preparation was conducted by the RNA-Seq pipeline v2.4 (FIMM Sequencing Core Laboratory) consisting of FastQC version 0.10.0 for quality control; reads were filtered for adaptor, rRNA and mtDNA sequences as well as homopolymer stretches using custom python scripts. Reads were aligned to human genome assembly (GRCh37.p7/hg19) with TopHat version 2.0.3^47^. Transcript quantification was conducted with cufflinks v 2.0.2^48^ with reference annotation (measured as FPKM) and gene expression was quantified by htseq-count^49^ (as raw read counts). Full details of library preparation, RNA-Seq and basic bioinformatics of raw data is provided in Supplementary Methods.

### Taqman RT-qPCR

Gene expression was quantitated by singleplex RT-qPCR of the target gene sequence using pre-made TaqMan Gene Expression Assays (Applied Biosystems, Life Technologies; Supplementary Table 5). In all experiments, a housekeeping gene *Ubiquitin C* (*UBC*) was used as reference gene. cDNA was synthesized from 1 μg total RNA according to the manufacturer’s instructions (SuperScript III Reverse Transcriptase, Life Technologies). All qPCR reactions were performed in triplicate in 384 micro-well plates in ABI 7900HT Real-time PCR system (Applied Biosystems, Life Technologies) using HOT FIREPol^®^ Probe qPCR Mix (Solis BioDyne, Tartu, Estonia). Full experimental details of the Taqman RT-qPCR are provided in Supplementary Methods.

### Statistical analysis

Differential expression in RNA-Seq data was tested using DESeq^50^ and DESeq2^51^ packages for R^52^. Read counts from htseq-count were used as input and built-in normalization algorithms of DESeq and DESeq2 were used. Outlier detection and handling was performed using the default method in DESeq. In DESeq2 outliers were replaced using the *replace Outliers With Trimmed Mean* function with default Cook’s distance cutoff. Statistical testing indicated that the two software packages, DESeq and DESeq2 differ substantially for their sensitivity and robustness in assessment of differential expression. Compared to the seminal DESeq package, analysis with the more recently developed DESeq2 programme produced a markedly higher number of significant results for all conducted differential expression tests with our data (Supplementary Table S1). Thus, in the current study a more stringent level of significance was imposed on the test results of DESeq2. A gene was considered as differentially expressed, when the statistical tests simultaneously satisfied the following empirically set thresholds: FDR < 0.1 for DESeq and FDR <0.05 for DESeq2. Genes with mean normalized expression <50 reads in all samples (n = 39425 DESeq; n = 39345 DESeq2) were considered as a transcriptional noise and filtered out from the analysis. No covariates were automatically included in the tested models. Instead, potential confounders (delivery mode, initiated labor activity, gestational age, gender, placental weight, birth weight/height, maternal pre-pregnancy BMI, weight gain, age and parity) were tested independently for the differential expression effect on all genes included into the analysis. For quantitative variables the samples were divided at the median value of the parameter. Gene set enrichment analysis was performed using g:Profiler^21^. Enrichment was tested for categories related to Gene Ontology, KEGG pathways and transcription factor (TF) regulatory motifs. Statistical testing, principal component analysis (PCA) and hierarchical clustering (Pearson correlation as the distance function) was performed in R. Statistical analyses for RT-qPCR results were performed using statistical package STATA version 13.1. Significance of RT-qPCR measurements among the study groups was assessed by Wilcoxon test. FDR was calculated according to Benjamini and Hochberg^53^. Additional details of the statistical analysis are provided in Supplementary Methods.

## Additional Information

**How to cite this article**: Sõber, S. *et al*. Extensive shift in placental transcriptome profile in preeclampsia and placental origin of adverse pregnancy outcomes. *Sci. Rep*. **5**, 13336; doi: 10.1038/srep13336 (2015).

## Supplementary Material

Supplementary Information

Supplementary Data S1

Supplementary Data S2

Supplementary Data S3

## Figures and Tables

**Table 1:** Mean (standard deviation) of maternal and newborn characteristics of samples used for the RNA-Seq and Taqman RT-qPCR experiments.

	Normal term	SGA	LGA	PE	GD
A. Individuals for RNA-Seq (n = 8/group)					
Maternal age (years)	29.3 ± 7.85	24.5 ± 3.51	30.9 ± 5.06	27.4 ± 7.39	30.3 ± 5.15
Maternal height (cm)	164.5 ± 5.34	165.4 ± 6.23	168.4 ± 6.39	168.8 ± 3.85	166.1 ± 5.28
Pre-pregnancy BMI (kg/m^2^	23.9 ± 3.77	20.6 ± 2.92	24.83 ± 4.61	26.3 ± 5.00	26.3 ± 7.85
Gestational weight gain (kg)	17.2 ± 3.52	13.3 ± 3.47*	21.7 ± 7.25	10.9 ± 3.36*	14.8 ± 5.76
Nulliparity (n)	3	7*	2	6	3
Smokers during pregnancy (n)	2	2	0	2	1
Gestational age at birth (days)	278.6 ± 11.5	272 ± 9.46	281.6 ± 4.41	266.1 ± 3.94*	275.5 ± 5.98
Labor activity (yes/no)	5/3	7/1	3/5	2/6	4/4
Delivery mode (vaginal/c-sect)	5/3	6/2	3/5	2/6	3/5
Paternal age (years)	31.8 ± 5.99	27.14 ± 5.64	35.8 ± 8.28	32.8 ± 8.94	33.5 ± 7.05
Paternal BMI (kg/m^2^)	25.1 ± 3.83	22.7 ± 2.04	29.9 ± 5.65	28.8 ± 6.34	27.8 ± 4.34
Newborn weight (g)	3703 ± 392	2442 ± 235*	4726 ± 208*	2794 ± 488*	4269 ± 238*
Newborn length (cm)	51.3 ± 1.89	46.3 ± 1.04*	53.4 ± 1.18*	47.6 ± 1.51*	52.4 ± 1.30
Ponderal index (g/cm^3^)	2.74 ± 0.27	2.48 ± 0.29*	3.11 ± 0.26*	2.57 ± 0.29	2.98 ± 0.27
IUGR (n)	0	5	0	4	0
Newborn sex (F/M)	3/5	5/3	4/4	4/4	5/3
Placental weight (g)	571.3 ± 115.8	397.5 ± 89.9*	816.2 ± 116.0*	476.9 ± 119.9	658.1 ± 185.2
B. Individuals for Taqman RT-qPCR experiments (n = 24/group)					
Maternal age (years)	29.5 ± 6.53	26.7 ± 5.29	30.6 ± 6.57	27.3 ± 5.15	31.6 ± 6.09
Maternal height (cm)	166.2 ± 4.94	166.3 ± 5.73	168.4 ± 6.72	167.6 ± 5.68	166.5 ± 6.35
Pre-pregnancy BMI (kg/m^2^	23.2 ± 3.53	21.1 ± 2.39*	25.6 ± 5.67	24.0 ± 4.39	25.8 ± 6.50
Gestational weight gain (kg)	15.7 ± 4.74	12.7 ± 3.00*	18.3 ± 6.11	15.0 ± 5.38	15.5 ± 5.51
Nulliparity (n)	9	14	10	17*	8
Smokers during pregnancy (n)	4	6	0*	3	5
Gestational age at birth (days)	274.7 ± 9.94	270.7 ± 9.40	282.0 ± 6.15*	261.7 ± 13.0*	274.6 ± 6.38
Delivery mode (vaginal/c-sect)	17/7	15/9	14/10	6/18*	14/10
Paternal age (years)	32.3 ± 5.32	28.3 ± 6.01*	34.2 ± 7.81	30.6 ± 6.56	33.5 ± 6.51
Paternal BMI (kg/m^2^)	27.3± 4.11	24.6 ± 3.10*	27.8 ± 4.60	26.6 ± 4.78	26.5 ± 4.11
Newborn weight (g)	3568 ± 419	2416 ± 273*	4824.5 ± 280*	2702 ± 611*	4110 ± 520*
Newborn length (cm)	50.9 ± 1.86	46.1 ± 1.71*	53.7 ± 1.70*	47.1 ± 3.22*	52.0 ± 1.52*
Ponderal index (g/cm^3^)	2.70 ± 0.304	2.47 ± 0.27*	3.12 ± 0.29*	2.54 ± 0.257	2.91 ± 0.25*
IUGR (n)	0	17	0	8	0
Newborn sex (F/M)	12/12	13/11	12/12	14/10	12/12
Placental weight (g)	550 ± 114.8	413.3 ± 82.3*	807.5 ± 141.3*	469 ± 115.6*	677.8 ± 152.5*

Data are given as arithmetic mean ± standard deviation, except where indicated differently.

Nulliparity refers to no previous childbirth; normal term indicates uncomplicated pregnancies resulting with the birth of a newborn with appropriate-for-gestational age; Ponderal index is a ratio of body weight to length: [weight (in g) x 100] ÷ [length (in cm)]^3^; BMI, body mass index; c-sect, caesarean section; F, female; GD, gestational diabetes; LGA, large-for-gestational age (birth weight > 90^th^ percentile); M, male; PE, preeclampsia; SGA, small-for-gestational age (birth weight < 10^th^ percentile); IUGR, intrauterine growth restriction.

^*^P value < 0.05 compared to normal term pregnancy group, Student’s T test (for quantitative variables) or χ^2^ test (for binary variables).

**Table 2:** Differentially expressed placental genes affected by confounding variables.

Variable^a^	Differentially expressed genes (FDR < 0.05)
Delivery-specific variables	
Delivery mode: vaginal delivery or caesarian section	*ZBTB16, KLF9, APOLD1, FKBP5, TSC22D3, ID4, BMP2, FAM107A, MAP1B, THBS1, IL18R1, LDB3, PER1, NIPA1, MYC, CEBPD, DDIT4, GADD45B, DGKG, SDPR, RP11-381O7.3, IGSF8, KCNK3, SLC41A2, PITPNC1, EVA1C, IRAK3*
Labor onset	*ZBTB16, KLF9, APOLD1, TSC22D3, FKBP5, JPH2, ID4, BMP2, MAP1B, CEBPD, IL18R1, IGSF8, THBS1, FAM43A, PADI2, PER1, ATOH8, GADD45B, DDIT4, PITPNC1, TMEM132C, PREX1, MYC, FAM107A, QRSL1P1, HPCAL1, MCF2L, LDB3*
Fetal and pregnancy-specific variables	
Offspring gender	*KDM5D, DDX3Y, ZFY, PCDH11Y, PRKY, USP9Y, RPS4Y1, TXLNG2P, UTY, EIF1AY, PSMA6P1, XIST, LINC00278, TTTY15, HDHD1, CD99, KDM6A, GYG2P1, KDM5C, STS*
Placenta weight	*LEP, HTRA4, TET3, RP11-465K1.2, PLIN2, SH3BP5, ARHGEF4, KRT19, QPCT, MID1, WDR86, LYN, WDR86-AS1, C8orf58, SASH1, MLPH*
Birth weight	*SASH1, NRIP1, ARNT2, RP11-465K1.2, TRIM14, LEP, AFAP1, LEPREL1, CTD-2383M3.1, ARHGAP42*
Gestational age	*SASH1, LEP, NRIP1, AF127577.11*
Birth length	0
Maternal variables: pre-pregnancy BMI, gestational weight gain, age, parity^b^	
	0

^a^For quantitative variables the sample was divided into two groups at the median value of the variable; differential expression was assessed using DESeq^50^.

^b^Number of previous deliveries.
