# Supplementary material for: Extensive shift in placental transcriptome profile in preeclampsia and placental origin of adverse pregnancy outcomes
Source: Sci Rep. 2015 Aug 13;5:13336. doi: 10.1038/srep13336 (PMC4542630; doi:10.1038/srep13336)
Supplement: Supplementary Information [file srep13336-s1.pdf]

Supplementary Information

Extensive shift in placental transcriptome profile in preeclampsia and placental origin of adverse pregnancy outcomes

Siim Sõber[1]*, Mario Reiman[1], Triin Kikas[1], Kristiina Rull[1,2,3], Rain Inno[1], Pille Vaas[2,3], Pille Teesalu[2,3], Jesus M. Lopez Marti [4], Pirkko Mattila[4], Maris Laan[1]*

[1]Human Molecular Genetics Research Group, Institute of Molecular and Cell Biology, University of Tartu, Tartu 51010, Estonia

[2]Department of Obstetrics and Gynaecology, University of Tartu, Puusepa St. 8, Tartu 51014, Estonia

[3]Women's Clinic of Tartu University Hospital, Puusepa St. 8, Tartu 51014, Estonia

[4]The Institute for Molecular Medicine Finland (FIMM), Tukholmankatu 8, Helsinki FI-00014, Finland

*co-corresponding authors: siims@ut.ee, maris.laan@ut.ee

List of Supplementary Material:

Supplementary Methods.

Supplementary Figure S1. RNA-Seq metrics for the 40 analyzed placental tissue samples.

Supplementary Figure S2. *Xist* gene expression shown as normalized read count in the placental samples subjected to RNA-Seq.

Supplementary Figure S3. Expression profiles for the genes positioning among the top-20 most highly expressed placental protein-coding genes in a specific pregnancy complication(s), but not in normal pregnancy.

Supplementary Figure S4. Venn diagram for the shared genes among the top 200 highest ranked genes in differential expression testing in pregnancy complications with Preeclampsia group divided according to the presence of IUGR.

Supplementary Table S1. Numbers of genes identified as differentially expressed in pregnancy complications based on the RNA-Seq dataset.

Supplementary Table S2. Enriched functional categories among differentially expressed genes in preeclampsia.

Supplementary Table S3. Differential placental gene expression analysis by Taqman RT-qPCR in complicated compared to normal pregnancies (n=24/group).

Supplementary Table S4. Genes with an opposite direction of the placental gene expression fold change in the two groups of small-for-gestational-age (SGA) and large-for-gestational-age (LGA) newborns compared to normal pregnancy.

Supplementary Table S5. TaqMan probe sets used in RT-qPCR experiments.

Supplementary Data S1. Top 200 transcripts and protein genes with the highest measured gene expression in FPKM in the studied pregnancy outcomes (a separate .xls file).

Supplementary Data S2. Genes reaching statistical significance (FDR<0.1) in differential expression analysis of potential confounding variables implemented in DESeq (a separate .xls file).

Supplementary Data S3. Top 300 differential expression test results in DESeq and DESeq2 for normal pregnancy placental samples compared to PE, GD, SGA, LGA study groups, and compared to PE without IUGR and PE with IUGR subgroups. (a separate .xls file).

Supplementary Data S4. Functional profiling of upregulated (n=42) and downregulated (n=173) genes in preeclamptic placentas (a separate .xls file).

Supplementary Methods.

Library preparation, RNA-Seq and basic bioinformatics of raw data: Preparation of RNA-Seq sequencing libraries, sequencing of transcriptomes and basic bioinformatic processing of the raw sequencing data (quality control, read alignment and transcript and gene expression estimation) were performed at the Sequencing Core facility of Finnish institute of Molecular Medicine (FIMM), University of Helsinki, Finland. High quality DNA-free total RNA (5 µg) was used for depletion of ribosomal RNA (Ribo-Zero™ rRNA Removal Kit, Epicentre, Madison, WI, USA). The rRNA depleted RNA was purified (NucleoSpin® RNA Clean-up XS, Macherey-Nagel, Duren, Germany) and reverse transcribed to ds cDNA (SuperScript™ Double-Stranded cDNA Synthesis Kit, Life Technologies, Carlsbad, CA, USA). Random hexamers (New England BioLabs, Ipswich, MA, USA) were used for priming the first strand synthesis reaction and SPRI beads (Agencourt AMPure XP, Beckman Coulter, Brea, CA, USA) for purification of cDNA.

Nextera™ Technology (Illumina, San Diego, CA, USA) was used for preparation of RNA-Seq Libraries. In order to add the Illumina specific bridgePCR compatible sites as well as bar codes and enrich the library, limited-cycle PCR (5 cycles) was done according to instructions of Nextera system. SPRI beads were used for purification of the PCR-products and the library QC was evaluated by Agilent Bioanalyzer (Agilent Technologies, Santa Clara, CA, USA). C-Bot (TruSeq PE Cluster Kit v3, Illumina, San Diego, CA, USA) was used for cluster generation and Illumina HiSeq2000 platform (HiSeq TruSeq v3 reagent kit**)** for paired end sequencing with 2 x 46 bp read length. Each transcriptome library was loaded to occupy 1/3 of the lane capacity in a flow cell. Two samples (1 x PE; 1 x LGA) were sequenced separately at ½ lane capacity and 2 x 101 bp read length.

Initial data analysis and preparation was conducted by the RNA-Seq pipeline v2.4 (FIMM) consisting of FastQC version 0.10.0 (S. Andrews. FastQC (2011) <http://www.bioinformatics.babraham.ac.uk/projects/fastqc/> Date of access: 29/06/2015) for quality control; reads were filtered for adaptor, rRNA and mtDNA sequences as well as homopolymer stretches using custom python scripts; read alignment was performed with TopHat version 2.0.3[1] using bowtie version 0.12.7[2]; transcript quantification was conducted with cufflinks v 2.0.2[3] with reference annotation (measured as FPKM) and gene expression was quantified by htseq-count[4] (as raw read counts). Human genome assembly (GRCh37.p7/hg19) from Ensembl v67 was used as a reference. Read alignment to genomic regions was estimated with Picard v1.63 (http://picard.sourceforge.net).

Statistical analysis: Differential expression in RNA-Seq data was tested using DESeq[5] and DESeq2[6] packages for R[7]. Read counts from htseq-count were used as input (number of Ensembl v67 genes with read counts n = 53893). Genes with mean normalized expression < 50 reads in all samples (n = 39425 DESeq; n = 39345 DESeq2) were considered as a transcriptional noise and filtered out from the analysis. After the exclusion of genes with negligible placental transcript counts, 14468 and 14548 genes entered differential expression testing implemented in DESeq and DESeq2 packages, respectively. Built-in normalization algorithms of DESeq and DESeq2 were used. Outlier detection and handling was performed using the default method in DESeq. In DESeq2 outliers were replaced using the *replaceOutliersWithTrimmedMean* function with default Cook's distance cutoff. Statistical testing indicated that the two software packages, DESeq and DESeq2 differ substantially for their sensitivity and robustness in assessment of differential expression. Compared to the seminal DeSeq package, analysis with the more recently

developed DESeq2 programme produced a markedly higher number of significant results for all conducted differential expression tests with our data (**Supplementary Table S1**). Thus, in the current study a more stringent level of significance was imposed on the test results of DESeq2. A gene was considered as differentially expressed, when the statistical tests simultaneously satisfied the following empirically set thresholds: FDR<0.1 for DESeq and FDR<0.05 for DESeq2. No covariates were automatically included in the tested models. Instead, potential confounders (delivery mode, initiated labor activity, gestational age, gender, placental weight, birth weight/height, maternal pre-pregnancy BMI, weight gain, age and parity) were tested independently for the differential expression effect on all genes included into the analysis. For quantitative variables the samples were divided at the median value of the parameter. As the tested parameters confounded the expression level of a limited number of genes (**Supplementary Data S2**), no covariates were included into differential expression testing across whole-transcriptome in pregnancy complications.

Gene set enrichment analysis of identified genes with significant differential expression was performed using g:Profiler[8]. Enrichment was tested for categories related to Gene Ontology, KEGG pathways and transcription factor (TF) regulatory motifs. Statistical testing, principal component analysis (PCA) and hierarchical clustering (Pearson correlation as the distance function) was performed in R. To achieve s scaled expression value (Z-score), the gene expression levels were subjected to variance stabilizing transformation in DESeq and standardized by subtracting the mean expression across all samples from its value for a given sample and then dividing by the standard deviation across all the samples. Data on the enrichment of gene expression in the placenta compared to other tissues was derived from the publicly available Protein Atlas v12 database reporting RNA-Seq analysis across 27 human

tissues[9].

The statistical analyses for RT-qPCR results were performed using statistical package STATA version 13.1. Significance of RT-qPCR measurements among the study groups was assessed by Wilcoxon test (no adjustment for covariates). For the genes estimated to be affected by confounding factors based on RNA-Seq data, logistic regression model was applied incorporating placental weight (*LEP*, *TET3*) and gestational age (*LEP*) as covariates. FDR was calculated according to Benjamini and Hochberg[10].

Taqman RT-qPCR

For an independent experimental validation, gene expression of 48 protein-encoding genes and a non-coding transcript *ITPK1-AS* (belonging to sense-antisense pair with *ITPK1*) was analyzed using Taqman RT-qPCR gene expression assays (Applied Biosystems, Life Technologies; **Supplementary Table S5**). Majority of the assessed genes (n=43) were selected from the top-list of differential expression in PE and two genes *(STS*, *FAM65B*) due to significantly altered expression in GD compared to NORM (DESeq: FDR<0.1 and DESeq2: FDR<0.05; **Supplementary Data S3**). Genes with unavailable pre-designed Taqman assay or low placental expression (< 100 normalized read count) were not subjected to validation. As no genes from the analysis of LGA *vs* NORM and SGA *vs* NORM satisfied the criteria to be selected to Taqman RT-qPCR validation, alternative differential expression tests among the study groups were performed (data not shown). Three additional genes showing trend for altered expression were taken forward to Taqman RT-qPCR: *SLC16A3* (GD *vs* LGA) and *MYO7B*, *TET3* (SGA *vs* LGA samples). All genes analyzed for the differential placental expression in PE (n=45) were also assessed in SGA samples; all genes tested in GD placentas (n=5) were also analyzed in the LGA

group (**Supplementary Table S5**).

Gene expression was quantitated by singleplex RT-qPCR of the target gene sequence using pre-made TaqMan Gene Expression Assays (Applied Biosystems, Life Technologies, **Supplementary Table S5**). In all experiments, a housekeeping gene *Ubiquitin C* (*UBC*) was applied as an endogenous control. The generated RNA-Seq dataset revealed *UBC* as one of the most stably expressed housekeeping genes in the human placenta, affected neither by gestational complications nor confounding variables of the mother, fetus, pregnancy and delivery (data not shown).

cDNA was synthesized from 1 μg total RNA according to the manufacturer's instructions (SuperScript III Reverse Transcriptase, Invitrogen, Life Technologies). All qPCR reactions were performed in triplicate in 384 micro-well plates in ABI 7900HT Real-time PCR system (Applied Biosystems, Life Technologies) using HOT FIREPol® Probe qPCR Mix (Solis BioDyne, Tartu, Estonia) and TaqMan Gene Expression Assays. Negative controls lacked template inputs. RT-qPCR reactions were initially denatured at 95°C for 15 min, followed by 40 cycles of 15 s at 95°C and 1 min at 60°C. Amplification efficiency of TaqMan assays was 90-110%.

Relative mRNA expression values were determined by comparative C_T method as described previously (Applied Biosystems, Life Technologies)[11,12].

References.

1. Kim, D. *et al.* TopHat2: accurate alignment of transcriptomes in the presence of insertions, deletions and gene fusions. *Genome Biol.* **14,** R36 (2013).

2. Langmead, B., Trapnell, C., Pop, M. & Salzberg, S. L. Ultrafast and memory-efficient alignment of short DNA sequences to the human genome. *Genome Biol* **10,** R25 (2009).

3. Trapnell, C. *et al.* Transcript assembly and quantification by RNA-Seq reveals unannotated transcripts and isoform switching during cell differentiation. *Nat. Biotechnol.* **28,** 511–515 (2010).

4. Anders, S., Pyl, P. T. & Huber, W. *HTSeq A Python framework to work with high-throughput sequencing data*. *bioRxiv* (2014). doi:10.1101/002824

5. Anders, S. & Huber, W. Differential expression analysis for sequence count data. *Genome Biol* **11,** R106 (2010).

6. Love, M. I., Huber, W. & Anders, S. Moderated estimation of fold change and dispersion for RNA-seq data with DESeq2. *Genome Biol.* **15,** 550 (2014).

7. R Core Team. R: A Language and Environment for Statistical Computing. (2014). at <http://www.r-project.org> Date of access: 29/06/2015.

8. Reimand, J., Kull, M., Peterson, H., Hansen, J. & Vilo, J. G:Profiler-a web-based toolset for functional profiling of gene lists from large-scale experiments. *Nucleic Acids Res.* **35,** (2007).

9. Uhlen, M. *et al.* Towards a knowledge-based Human Protein Atlas. *Nat. Biotechnol.* **28,** 1248–1250 (2010).

10. Benjamini, Y. & Hochberg, Y. Controlling the False Discovery Rate: A Practical and Powerful Approach to Multiple Testing. *J. R. Stat. Soc. Ser. B* **57,** 289 – 300 (1995).

11. Uusküla, L. *et al.* Mid-Gestational Gene Expression Profile in Placenta and Link to Pregnancy Complications. *PLoS One* **7,** (2012).

12. Rull, K. *et al.* Increased placental expression and maternal serum levels of apoptosis-inducing TRAIL in recurrent miscarriage. *Placenta* **34,** 141–148 (2013).

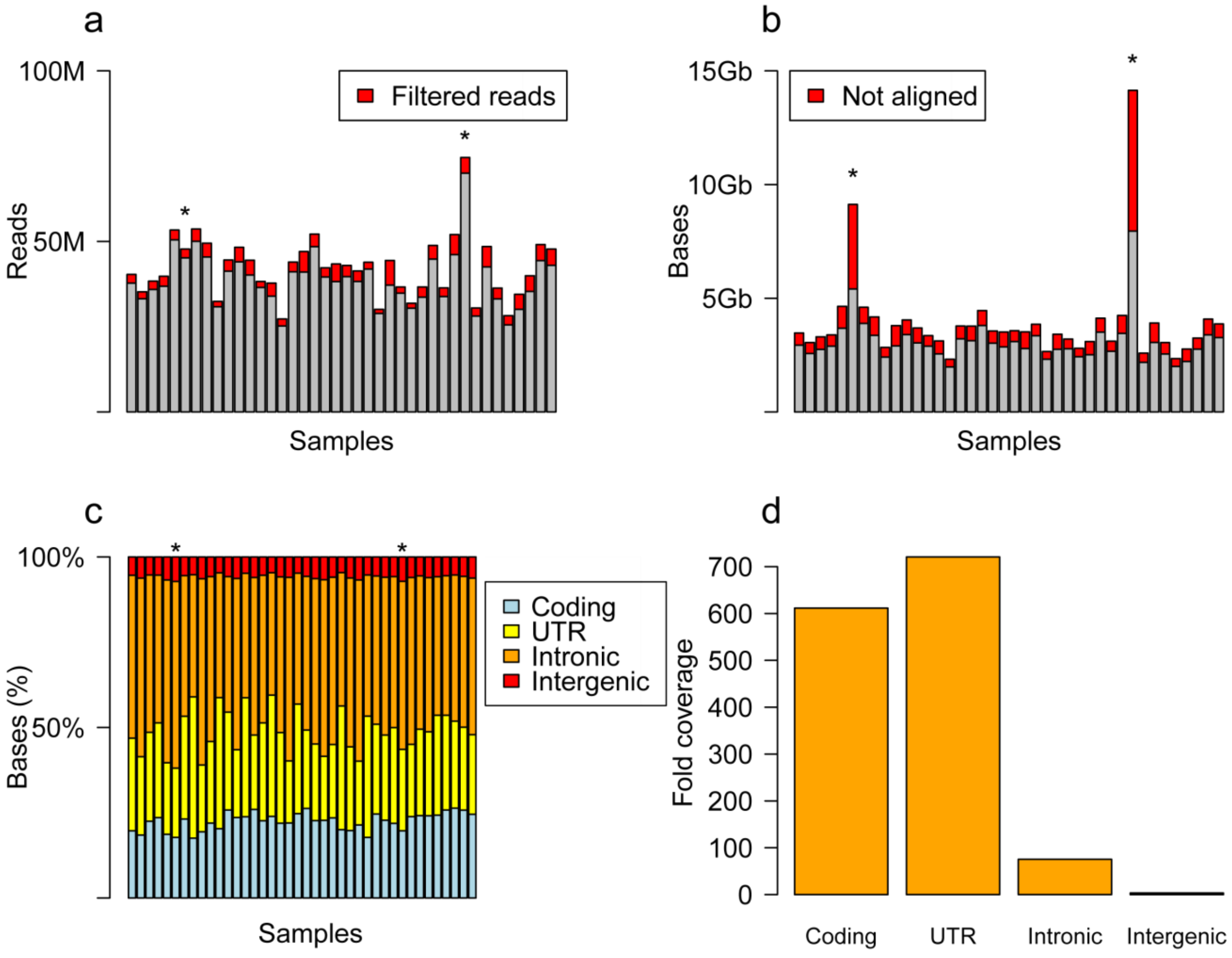

Supplementary Figure S1. RNA-Seq metrics for the 40 analyzed placental tissue samples. Majority (n=38) were subjected to 50-bp paired-end sequencing (Illumina HiSeq 2000), two samples were sequenced for 100+100 bp (indicated with *). **(a)** Total count of read pairs; excluded reads for not matching the filtering criteria are highlighted in red, representing rRNA, mtDNA or adaptor sequences and sequences containing homopolymer stretches. **(b)** Alignment efficiency; the bars represent the total length of all filtered reads per sample (in Gb) and the sequence fraction unmapped to the human genome reference (Ensembl v67; GRCh37.p7) is shown in red. **(c)** Proportions of aligned bases mapping to the functional genomic domains. (**d**) Sequence coverage of the functional genomic domains estimated for the full dataset across samples; expressed as the mean coverage per bp. *Coding*, protein coding; *UTR*, untranslated regions; *intronic*, introns of multi-exon genes; *intergenic*, regions not annotated as genes.

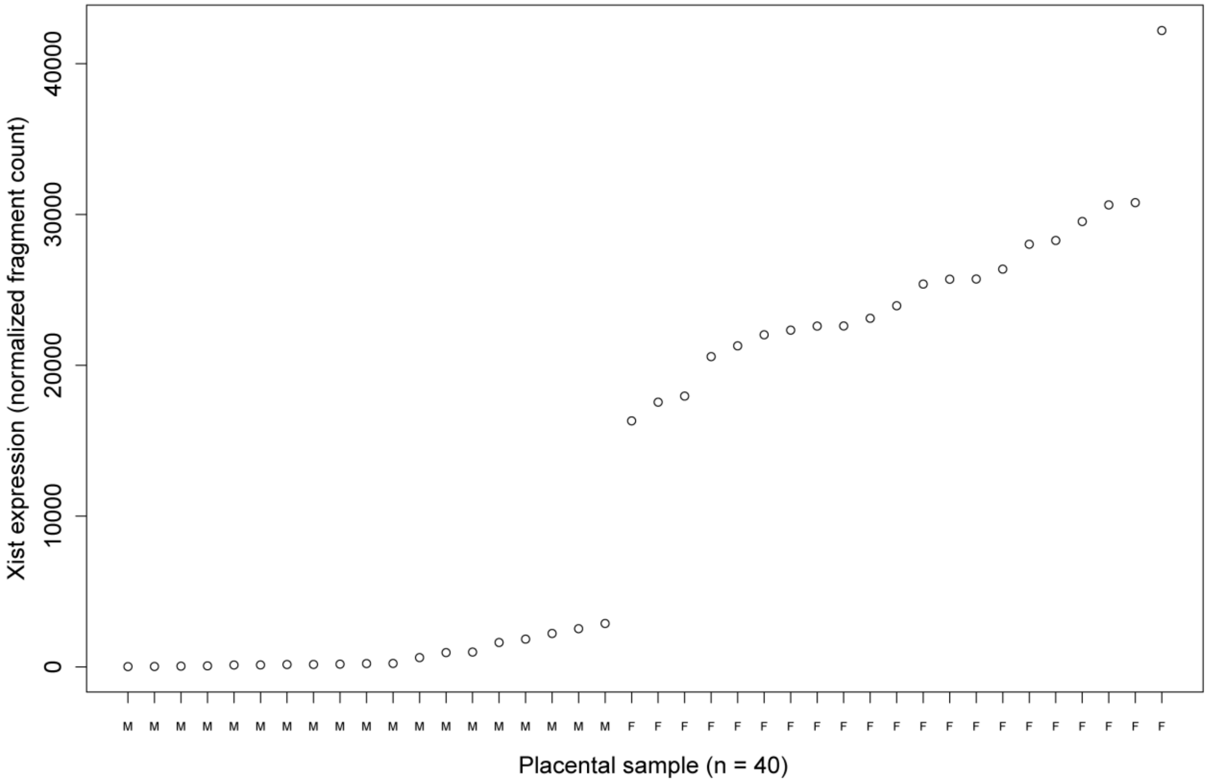

Supplementary Figure S2. *Xist* gene expression shown as normalized read count in the placental samples subjected to RNA-Seq. Samples (XY, n=19; XX, n=21) have been ordered by the level of gene expression.

F, female; M, male newborn; *Xist*, X-inactive specific transcript.

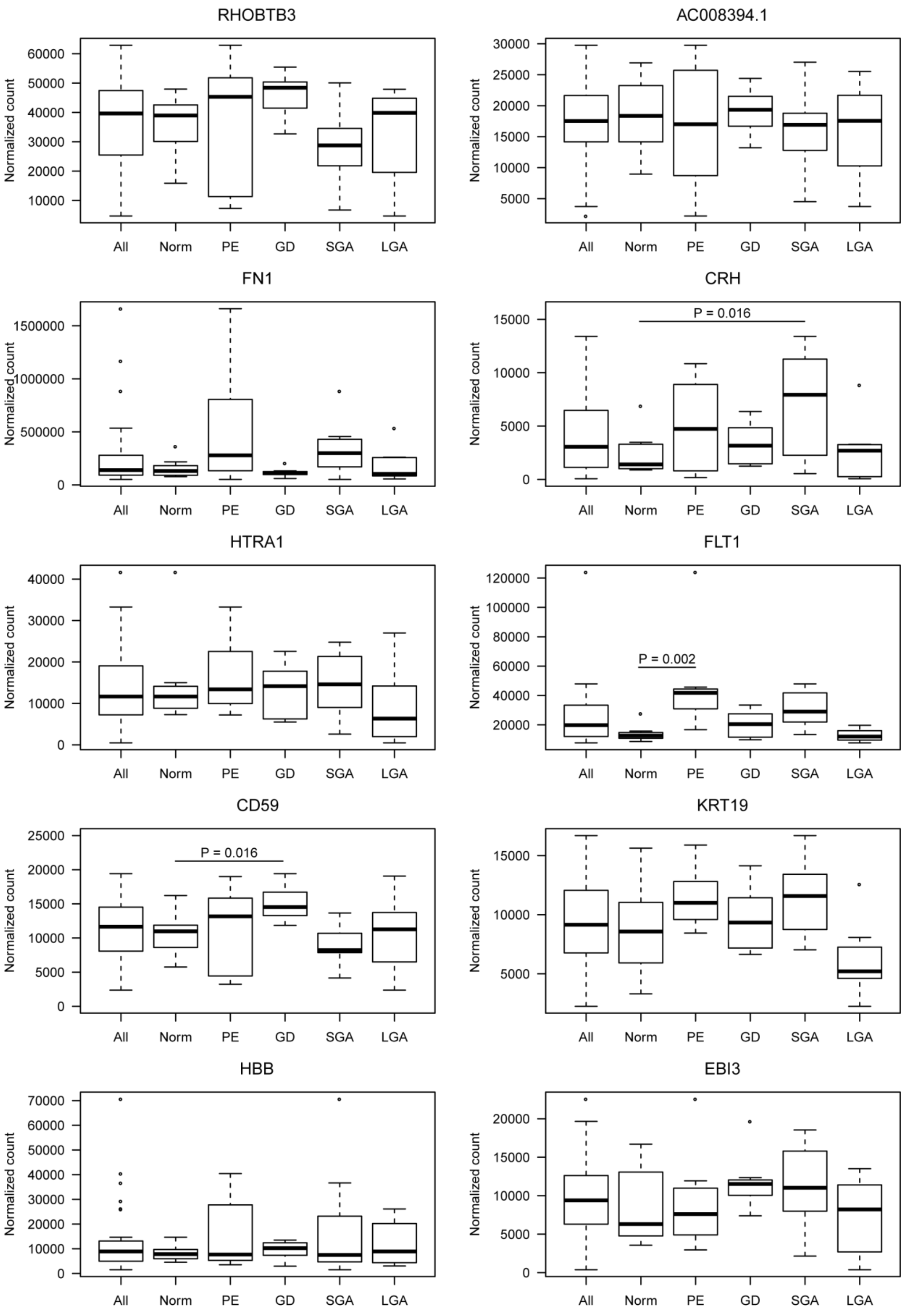
RHOBTB3
AC008394.1
FN1
CRH
P = 0.016
HTRA1
FLT1
P = 0.002
CD59
P = 0.016
KRT19
HBB
EBI3
Normalized count
All Norm PE GD SGA LGA

Supplementary Figure S3. Expression profiles for the genes positioning among the top-20 most highly expressed placental protein-coding genes in a specific pregnancy complication(s), but not in normal pregnancy (details in **Fig. 1**). Boxplots are based on the RNA-Seq data (normalized read counts) across all studied samples representing normal pregnancies (NORM) and adverse pregnancy outcomes (n=8/group).

GD, gestational diabetes; LGA, large-for-gestational-age newborns; SGA, small-for-gestational-age newborns; PE, preeclampsia; *AC008394.1*, annotated protein-coding transcript ENST00000445770.2; *CD59*, complement regulatory protein CD59; *CRH*, corticotropin releasing hormone; *EBI3*, Epstein-Barr virus induced 3; *FLT1*, fms-related tyrosine kinase 1, *FN1*, fibronectin 1; *HBB*, hemoglobin, beta; *HTRA1*, HtrA serine peptidase 1; *KRT19*, keratin 19; *RHOBTB3*, Rho-related BTB domain containing 3. *P*-values are from DESeq differential expression analysis.

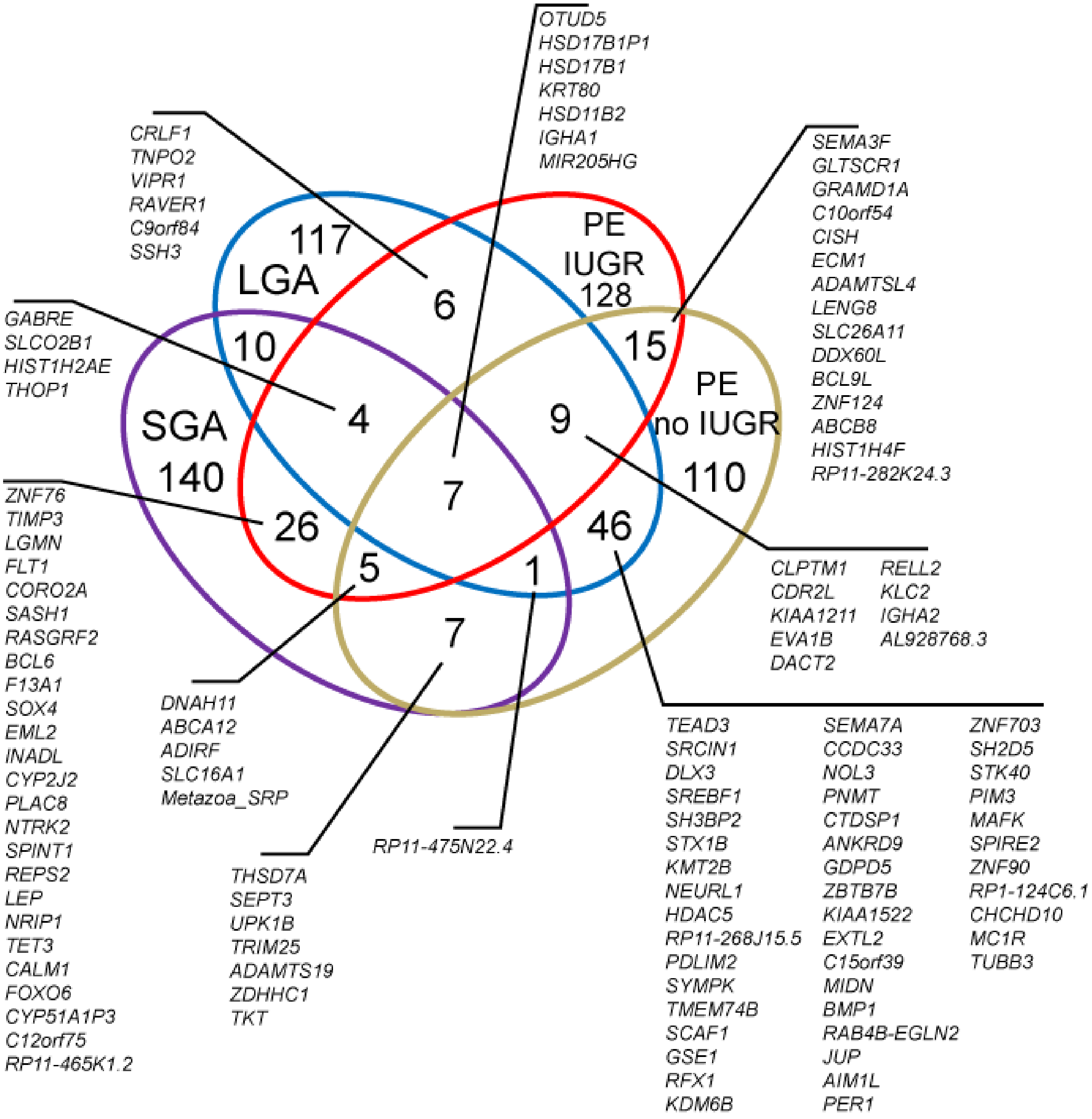

Supplementary Figure S4. Venn diagram for the shared genes among the top 200 highest ranked genes in differential expression testing in pregnancy complications with Preeclampsia group divided according to the presence of IUGR. The number and gene list in each intersection are given.

PE, preeclampsia, GD, gestational diabetes, SGA and LGA, small- and large-for-gestational-age newborns, IUGR – intrauterine growth restriction.

Supplementary Table S1. Numbers of genes identified as differentially expressed in pregnancy complications based on the RNA-Seq dataset.

Group[a]	FDR = 0.1			FDR = 0.05			Matching differential expression criteria[b]
	DESeq	DESeq2	Overlap	DESeq	DESeq2	Overlap	
**PE**	225	3485	220	125	2462	123	215
**GD**	9	372	4	2	108	0	4
**SGA**	3	58	2	3	26	2	2
**LGA**	5	49	1	5	15	1	1

[a] Differential expression was addressed using 8 case and 8 control (normal term) placental samples
[b] Genes were considered as differentially expressed if DESeq: FDR<0.1 and DESeq2: FDR<0.05
GD, gestational diabetes; LGA, pregnancies with large-for-gestational-age newborns; PE, preeclampsia; SGA, pregnancies with small-for-gestational-age newborns

Supplementary Table S2. Enriched functional categories among differentially expressed genes in preeclampsia.

			Gene enrichment[b]		
**Category**	**Term ID**	**DNA binding motif**	**n**	**%**	**P value**
**Upregulated genes (n=42[a])**					
Polyol biosynthetic process	GO:0046173	NA	3	8.60	3.04×10^{-2}
**Downregulated genes (n=173[a])**					
Extracellular matrix	GO:0031012	NA	14	8.40	1.76×10^{-3}
Proteinaceous extracellular matrix	GO:0005578	NA	13	7.80	2.46×10^{-3}
TF: AP2	TF:M00800_4	GSCCSCRGGCNRNRNN	115	69.3	8.14×10^{-9}
	TF:M00189_4	MKCCCSCNGGCG	78	47	7.88×10^{-4}
	TF:M00915_4	SNNNCCNCAGGCN	96	57.8	3.55×10^{-7}
TF: AP2alpha	TF:M00469_4	GCCNNNRGS	41	24.7	1.95×10^{-4}
TF: AP2gamma	TF:M00470_4	GCCYNNGGS	43	25.9	1.18×10^{-3}
TF: Egr	TF:M00807_4	GTGGGSGCRRS	46	27.7	2.21×10^{-2}
TF: ETF	TF:M00695_0	GVGGMGG	68	41	3.58×10^{-2}
TF: LRF	TF:M01100_4	VNNRMCCCC	140	84.3	5.96×10^{-9}
TF: MAZ	TF:M00649_0	GGGGAGGG	66	39.8	3.30×10^{-2}
TF: Sp1	TF:M00933_4	CCCCGCCCCN	94	56.6	8.23×10^{-6}
	TF:M00931_4	GGGGCGGGGC	90	54.2	4.33×10^{-5}
	TF:M00008_4	GGGGCGGGGT	109	65.7	1.91×10^{-5}
	TF:M00196_4	GGGGAGGG	95	57.2	1.92×10^{-6}
TF: VDR	TF:M00444_4	GGGKNARNRRGGWSA	125	75.3	2.80×10^{-3}

[a] Genes matching study criteria for significant differential expression (DESeq: FDR<0.1 and DESeq2: FDR < 0.05).
[b] Statistical analysis of gene enrichment in functional categories was implemented in gProfiler [10]. n, number of genes in the specific pathway within the tested gene list; %, fraction of analyzed genes in the pathway

Supplementary Table S3. Differential placental gene expression analysis by Taqman RT-qPCR in complicated compared to normal pregnancies (n=24/group).

	PE (N=24)				SGA (N=24)			
**Gene**	**FC**	**Concordant effects[a]**	**P value[b]**	**FDR[c]**	**FC**	**Concordant effects[a]**	**P value[b]**	**FDR[c]**
TMEM74B	0.473	+	1.00E-04	0.002	0.526	+	0.002	0.045
FLT1	2.578	+	1.00E-04	0.002	1.799	+	0.002	0.045
CDR2L	0.687	+	5.00E-04	0.005	0.707	+	0.003	0.045
IGHA1	0.512	+	3.00E-04	0.005	0.689	+	0.020	0.162
LEP	10.017	+	3.60E-04	0.003	3.081	+	0.009	0.085
HSD17B1	0.505	+	4.00E-04	0.005	0.739	+	0.126	0.421
MC1R	0.547	+	8.00E-04	0.006	0.771	+	0.242	0.494
DOT1L	0.667	+	1.50E-03	0.010	0.844	+	0.360	0.627
STX1B	0.580	+	0.004	0.021	0.911	+	0.718	0.900
GRAMD1A	0.581	+	0.009	0.040	0.695	+	0.217	0.494
ITPK1-AS1	0.696	+	0.009	0.040	0.790	+	0.120	0.421
ZNF469	0.652	+	0.009	0.040	0.737	+	0.475	0.770
SLC25A35	0.664	+	0.014	0.051	0.694	+	0.024	0.162
DLX4	0.662	+	0.013	0.051	0.738	+	0.071	0.301
GDPD5	0.647	+	0.019	0.056	0.651	+	0.050	0.262
TMEM8A	0.758	+	0.018	0.056	0.806	+	0.101	0.397
ITPK1	0.707	+	0.019	0.056	0.784	+	0.191	0.473
ADM	0.697	+	0.026	0.072	0.697	+	0.159	0.461
OTUD5	0.838	+	0.030	0.079	0.917	+	0.734	0.900
TET3	1.539	+	0.039	0.096	1.662	+	0.006	0.071
RELL2	0.529	+	0.041	0.097	0.643	+	0.242	0.494
HSD11B2	0.696	+	0.050	0.112	0.731	+	0.167	0.461
KIAA1211	0.638	+	0.070	0.142	1.135	-	0.496	0.777
RFX1	0.724	+	0.073	0.143	0.867	+	0.915	0.935
SH3PXD2A	1.580	+	0.080	0.150	1.428	+	0.024	0.162
DLX3	0.806	+	0.101	0.176	0.869	+	0.360	0.627
SLC26A11	0.834	+	0.112	0.189	0.920	+	0.915	0.935
KLC2	0.817	+	0.138	0.223	0.814	+	0.148	0.461
ABCA12	1.492	+	0.149	0.233	1.133	+	0.187	0.473
ECM1	0.809	+	0.209	0.316	0.880	+	0.882	0.935
AQPEP	1.589	+	0.275	0.403	0.907	-	0.395	0.662
SPINT1	0.865	+	0.312	0.445	2.310	-	0.832	0.930
LTBP3	0.969	+	0.343	0.474	0.912	+	0.766	0.900
BCL6	1.196	+	0.496	0.614	1.308	+	0.071	0.301
TEAD3	1.058	-	0.496	0.614	1.054	-	0.307	0.601
TMPRSS6	0.898	+	0.496	0.614	0.927	-	0.352	0.627
CSF3R	0.831	+	0.471	0.614	0.970	+	0.860	0.935

DACT2	0.885	+	0.509	0.614	0.857	+	0.580	0.802
MYO7B	1.210	+	0.523	0.614	0.869	-	0.655	0.879
SRCIN1	0.842	+	0.550	0.630	1.028	-	0.766	0.900
C10orf54	0.895	+	0.695	0.778	0.847	+	0.551	0.785
ADAMTSL4	1.070	-	0.742	0.810	1.062	-	0.551	0.785
SEMA3F	1.043	-	0.902	0.921	1.060	-	0.551	0.785
IPMK	1.102	+	0.902	0.921	1.034	+	0.798	0.915
DDX60L	1.048	+	0.951	0.951	0.852	-	0.225	0.494

	**GD (N = 24)**				**LGA (N = 24)**			
**Gene**	**FC**	**Concordant effects**[a]	**P value**[b]	**FDR**[c]	**FC**	**Concordant effects**[a]	**P value**[b]	**FDR**[c]
LEP	1.406	+	0.468	0.585	2.388	-	0.643	0.709
TET3	1.431	+	0.282	0.350	1.936	-	0.307	0.520
SLC16A3	1.462	+	0.013	0.065	1.090	-	0.278	0.520
STS	1.032	+	0.757	0.760	0.902	-	0.621	0.670
FAM65B	1.300	+	0.248	0.350	1.167	-	0.665	0.670

FC - fold change; PE - preeclampsia, GD - gestational diabetes, SGA - small for gestational age, LGA - large for gestational age.

[a] - Concordant effect direction in normal group compared to complication group from RNA-Seq and RT-qPCR experiments

[b] - Wilcoxon test

[c] - FDR was calculated according to Benjamini and Hochberg

Supplementary Table S4. Genes with an opposite direction of the placental gene expression fold change in the two groups of small-for-gestational-age (SGA) and large-for-gestational-age (LGA) newborns compared to normal pregnancy.

Ensembl ID	Name	Fold change in SGA	Fold change in LGA	Type
ENSG00000132613	MTSS1L	1.570	0.597	protein_coding
ENSG00000147872	PLIN2	1.530	0.504	protein_coding
ENSG00000169495	HTRA4	2.382	0.678	protein_coding
ENSG00000089847	ANKRD24	1.550	0.684	protein_coding
ENSG00000185112	FAM43A	1.503	0.705	protein_coding
ENSG00000183779	ZNF703	1.404	0.513	protein_coding
ENSG00000073060	SCARB1	1.401	0.708	protein_coding
ENSG00000172379	ARNT2	1.872	0.718	protein_coding
ENSG00000107518	ATRNL1	0.590	1.387	protein_coding
ENSG00000113916	BCL6	1.714	0.723	protein_coding
ENSG00000169994	MYO7B	2.080	0.741	protein_coding
ENSG00000107819	SFXN3	1.341	0.575	protein_coding
ENSG00000105137	SYDE1	1.336	0.562	protein_coding
ENSG00000241852	C8orf58	1.721	0.762	protein_coding
ENSG00000113946	CLDN16	0.397	1.311	protein_coding
ENSG00000116883	RP11-268J15.5	1.311	0.518	protein_coding
ENSG00000174697	LEP	12.275	0.766	protein_coding
ENSG00000107957	SH3PXD2A	1.784	0.768	protein_coding
ENSG00000254087	LYN	1.585	0.786	protein_coding
ENSG00000090776	EFNB1	1.269	0.667	protein_coding
ENSG00000144791	LIMD1	1.266	0.676	protein_coding
ENSG00000171097	CCBL1	1.693	0.835	protein_coding
ENSG00000154262	ABCA6	0.526	1.195	protein_coding
ENSG00000107954	NEURL1	1.183	0.641	protein_coding
ENSG00000163286	ALPPL2	1.176	0.393	protein_coding
ENSG00000214193	SH3D21	1.174	0.588	protein_coding
ENSG00000166866	MYO1A	0.356	1.170	protein_coding
ENSG00000187605	TET3	1.778	0.855	protein_coding
ENSG00000113719	ERGIC1	1.541	0.857	protein_coding
ENSG00000139971	C14orf37	1.813	0.859	protein_coding
ENSG00000214357	NEURL1B	2.055	0.866	protein_coding
ENSG00000087266	SH3BP2	1.153	0.575	protein_coding
ENSG00000232986	RP11-179A7.2	0.539	1.149	lincRNA
ENSG00000131037	EPS8L1	1.142	0.561	protein_coding
ENSG00000196975	ANXA4	1.141	0.708	protein_coding

ENSG00000162704	ARPC5	0.668	1.139	protein_coding
ENSG00000125503	PPP1R12C	1.128	0.676	protein_coding
ENSG00000120913	PDLIM2	1.118	0.665	protein_coding
ENSG00000243156	MICAL3	1.711	0.896	protein_coding
ENSG00000064687	ABCA7	1.746	0.903	protein_coding
ENSG00000165323	FAT3	0.904	1.947	protein_coding
ENSG00000093100	XXbac-B461K10.4	2.241	0.907	processed_transcript
ENSG00000166592	RRAD	2.340	0.919	protein_coding
ENSG00000131149	GSE1	1.084	0.663	protein_coding
ENSG00000140481	CCDC33	1.078	0.538	protein_coding
ENSG00000178209	PLEC	1.075	0.651	protein_coding
ENSG00000143036	SLC44A3	1.768	0.931	protein_coding
ENSG00000162747	FCGR3B	1.073	0.480	protein_coding
ENSG00000185842	DNAH14	0.932	1.567	protein_coding
ENSG00000102755	FLT1	2.242	0.932	protein_coding
ENSG00000141098	GFOD2	1.063	0.685	protein_coding
ENSG00000162512	SDC3	1.055	0.668	protein_coding
ENSG00000185215	TNFAIP2	2.079	0.954	protein_coding
ENSG00000230490	RP11-141M1.3	0.531	1.036	lincRNA
ENSG00000136235	GPNMB	0.550	1.034	protein_coding
ENSG00000197121	PGAP1	0.972	1.468	protein_coding
ENSG00000246859	STARD4-AS1	0.975	1.486	antisense
ENSG00000152078	TMEM56	0.976	1.744	protein_coding
ENSG00000235162	C12orf75	2.205	0.976	protein_coding
ENSG00000144579	CTDSP1	1.021	0.688	protein_coding
ENSG00000246090	RP11-696N14.1	0.983	1.605	antisense
ENSG00000179094	PER1	1.009	0.621	protein_coding
ENSG00000198668	CALM1	1.486	0.992	protein_coding
ENSG00000072310	SREBF1	1.003	0.660	protein_coding
ENSG00000106991	ENG	1.874	0.997	protein_coding

Supplementary Table S5. TaqMan probe sets used in RT-qPCR experiments.

Genes analyzed by Taqman RT-qPCR			Analyzed clinical subgroups				
Symbol	Gene Name	Taqman Assay ID	Norm	PE	SGA	GD	LGA
UBC (reference)	**ubiquitin C**	**Hs00824723_m1**	**+**	**+**	**+**	**+**	**+**
ABCA12	ATP-binding cassette; sub-family A member 12	Hs00292421_m1	+	+	+		
ADAMTSL4	ADAMTS-like 4	Hs00417524_m1	+	+	+		
ADM	adrenomedullin	Hs00969450_g1	+	+	+		
AQPEP	laeverin	Hs01060572_m1	+	+	+		
BCL6	B-cell CLL/lymphoma 6	Hs00153368_m1	+	+	+		
C10orf54	chr 10 open reading frame 54	Hs00735289_m1	+	+	+		
CDR2L	cerebellar degeneration-related protein 2-like	Hs00412746_m1	+	+	+		
CSF3R	colony stimulating factor 3 receptor (granulocyte)	Hs00167918_m1	+	+	+		
DACT2	dapper; antagonist of beta-catenin; homolog 2 (Xenopus laevis)	Hs00915740_m1	+	+	+		
DDX60L	DEAD (Asp-Glu-Ala-Asp) box polypeptide 60-like	Hs01016279_m1	+	+	+		
DLX3	distal-less homeobox 3	Hs00270938_m1	+	+	+		
DLX4	distal-less homeobox 4	Hs00231080_m1	+	+	+		
DOT1L	DOT1-like; histone H3 methyltransferase (S. cerevisiae)	Hs01588547_m1	+	+	+		
ECM1	extracellular matrix protein 1	Hs00189435_m1	+	+	+		
FAM65B	family with sequence similarity 65; member B	Hs00210599_m1	+			+	+
FLT1	fms-related tyrosine kinase 1 (vascular endothelial growth factor/vascular permeability factor receptor)	Hs01052961_m1	+	+	+		
GDPD5	glycerophosphodiester phosphodiesterase domain containing 5	Hs00229270_m1	+	+	+		

GRAMD1A	GRAM domain containing 1A	Hs00385157_m1	+	+	+		
HSD11B2[a]	hydroxysteroid (11-beta) dehydrogenase 2	Hs00388669_m1	+	+	+		
HSD11B2	hydroxysteroid (11-beta) dehydrogenase 2	Hs00930759_g1	+	+	+		
HSD17B1	hydroxysteroid (17-beta) dehydrogenase 1	Hs00166219_g1	+	+	+		
IGHA1	immunoglobulin heavy constant alpha 1	Hs00733892_m1	+	+	+		
ITPK1	inositol-tetrakisphosphate 1-kinase	Hs00356546_m1	+	+	+		
ITPK1-AS1	ITPK1 antisense RNA 1 (non-protein coding)	Hs01053867_s1	+	+	+		
KIAA1211	uncharacterized protein;KIAA1211	Hs00393402_m1	+	+	+		
KLC2	kinesin light chain 2	Hs00224491_m1	+	+	+		
LEP	leptin	Hs00174877_m1	+	+	+	+	+
LTBP3	latent transforming growth factor beta binding protein 3	Hs01105746_m1	+	+	+		
MC1R	melanocortin 1 receptor (alpha melanocyte stimulating hormone receptor)	Hs00267168_s1	+	+	+		
MIR205HG	MIR205 host gene (non-protein coding)	Hs03405498_m1	+	+	+		
MYO7B	myosin VIIB	Hs00400099_m1	+	+	+		
OTUD5	OTU domain containing 5	Hs00380113_m1	+	+	+		
RELL2	RELT-like 2	Hs00946747_g1	+	+	+		
RFX1	regulatory factor X; 1 (influences HLA class II expression)	Hs00172561_m1	+	+	+		
SEMA3F	sema domain; immunoglobulin domain (Ig); short basic domain; secreted; (semaphorin) 3F	Hs00188273_m1	+	+	+		
SH3PXD2A	SH3 and PX domains 2A	Hs00206037_m1	+	+	+		
SLC16A3	solute carrier family 16; member 3 (monocarboxylic acid transporter 4)	Hs00358829_m1	+			+	+
SLC25A35	solute carrier family 25; member 35	Hs01390367_m1	+	+	+		
SLC26A11	solute carrier family 26; member 11	Hs00543412_m1	+	+	+		
SPINT1	serine peptidase inhibitor; Kunitz type 1	Hs00173678_m1	+	+	+		
SRCIN1	SRC kinase signaling inhibitor 1	Hs00382426_m1	+	+	+		

STS	steroid sulfatase (microsomal); isozyme S	Hs00996676_m1	+			+	+
STX1B	syntaxin 1B	Hs01041315_m1	+	+	+		
TEAD3	TEA domain family member 3	Hs00243231_m1	+	+	+		
TET3	tet methylcytosine dioxygenase 3	Hs00379125_m1	+	+	+	+	+
TMEM74B	transmembrane protein 74B	Hs00217957_m1	+	+	+		
TMEM8A	transmembrane protein 8A	Hs00430489_m1	+	+	+		
TMPRSS6	transmembrane protease; serine 6	Hs00542184_m1	+	+	+		

[a] two alternative probe set were used due to low efficiency of PCR amplification (<90%)
NORM, uncomplicated pregnancies resulting in the birth of newborn with normal birth weight; SGA, pregnancies resulted in the birth of baby born as small-for-gestational age,<10th centile; LGA, pregnancies resulted in the birth of baby born as large-for-gestational age, >10th centile; PE, pregnancies complicated with severe preeclampsia; GD, pregnancies complicated with gestational diabetes
The weight centiles for defining SGA and LGA were calculated on the basis of data from Estonian Medical Birth Registry (Ref: Karro H, Rahu M, Gornoi K, Baburin A (1997) *Eesti Arst* (4):299-303 [in Estonian])
